# Gene Expression Profiling of Shoot-Derived Calli from Adult Radiata Pine and Zygotic Embryo-Derived Embryonal Masses

**DOI:** 10.1371/journal.pone.0128679

**Published:** 2015-06-03

**Authors:** O. Garcia-Mendiguren, I. A. Montalbán, D. Stewart, P. Moncaleán, K. Klimaszewska, R. G. Rutledge

**Affiliations:** 1 Neiker-Tecnalia, Campus Agroalimentario de Arkaute, Vitoria-Gazteiz, Spain; 2 Natural Resources Canada, Canadian Forest Service, Laurentian Forestry Centre, Québec, Canada; United States Department of Agriculture, UNITED STATES

## Abstract

**Background:**

Although somatic embryogenesis has an unprecedented potential for large-scale clonal propagation of conifers, the ability to efficiently induce the embryonal cultures required for somatic embryo production has long been a challenge. Furthermore, because early stage zygotic embryos remain the only responsive explants for pines, it is not possible to clone individual trees from vegetative explants at a commercial scale. This is of particular interest for adult trees because many elite characteristics only become apparent following sexual maturation.

**Findings:**

Shoot explants collected from adult radiata pine trees were cultured on four induction media differing in plant growth regulator composition, either directly after collection or from *in vitro*-generated axillary shoots. Six callus lines were selected for microscopic examination, which failed to reveal any embryonal masses (EM). qPCR expression profiling of five of these lines indicated that explant type influenced the absolute level of gene expression, but not the type of genes that were expressed. The analysis, which also included three EM lines induced from immature zygotic embryos, encompassed five categories of genes reflective of metabolic, mitotic and meristematic activity, along with putative markers of embryogenicity. Culture medium was found to have no significant impact on gene expression, although differences specific to the explant’s origin were apparent. Expression of transcriptional factors associated with vegetative meristems further suggested that all of the callus lines possessed a substantive vegetative character. Most notable, however, was that they all also expressed a putative embryogenic marker (LEC1).

**Conclusions:**

While limited in scope, these results illustrate the utility of expression profiling for characterizing tissues in culture. For example, although the biological significance of LEC1 expression is unclear, it does present the possibility that these callus lines possess some level of embryogenic character. Additionally, expression of vegetative meristem markers is consistent with their vegetative origin, as are differences in expression patterns as compared with EM.

## Introduction

Modern forest management relies extensively on breeding and reforestation programs to support both the sustainability of forest productivity and conservation of natural forests, with the expectation that plantation forestry will play a major role [1]. As such, vegetative propagation has become an integral part of many tree improvement programs, primarily due to its ability to clonally propagate elite genotypes. This is indicative of the widely held belief that cloning individual trees allows large genetic gains to be achieved within a single selection cycle, and of interest in developing the capability to clone adult trees because many elite characteristics only become established after sexual maturation [2–5].

Although juvenile conifer trees can be cloned through rooted cuttings, as trees mature they become increasingly unresponsive. Indeed, loss of juvenility in conifers is particularly persistent and difficult to reverse [5,6]. Nevertheless, a number of reports have described successful production of propagules from adult conifers using vegetative propagation. These include *in vitro* serial micrografting followed by rooting of grafted shoots of European larch (*Larix decidua* Mill.) [7], and rooting of axillary shoots of adult *Pinus pinaster* [8], *P*. *sylvestris* [9], and *P*. *radiata* [10]. However, vegetative propagation through rooting of shoots presents many limitations, the most prominent being the limited number of propagules that can be generated for reforestation programs, which have historically relied on large-scale production of conifer seedlings from seed.

Somatic embryogenesis (SE) provides an alternative approach that is in many ways analogous to large-scale seedling production from seed [2,11]. In addition to allowing the exploitation of existing reforestation infrastructure, SE provides the capability for large-scale clonal propagation, in that somatic embryos produced from an individual EM line (genotype) are genetically identical. Combined with the ability to cryopreserve vast numbers of EM lines, SE has the potential to generate an unlimited numbers of somatic seedlings, albeit with one major limitation.

Notwithstanding the potential of SE for clonal propagation, the recalcitrance of vegetative explants to generate EM (SE induction) has precluded the ability to clone individual trees. Additionally, while young needles from 1-year-old Norway spruce (*Picea abies* (l.) Karst.) have been found to have limited responsiveness to SE induction [12], this responsiveness is lost as donor trees age, presenting an additional hurdle to cloning mature trees via SE [4,5,13].

A prominent exception is a group of somatic embryo-derived trees generated from a single white spruce (*P*. *glauca* (Moench) Voss) EM line, in which primordial shoots have remained responsive to SE induction for over a decade, and have continued to be responsive even after reaching sexual maturity [14]. While the mechanisms underpinning this persistent responsiveness have yet to be resolved, transcriptome analysis has suggested that a moderate response to the stress associated with the SE induction treatment could play an important role [15]. Indeed, it has been well established that stress response is a key element for SE induction in a wide variety of plant species, although the underlying molecular pathways remain largely unknown [16,17]. Developing more effective SE induction protocols is further exacerbated by a general lack of understanding of even the most fundamental molecular mechanisms associated with the formation and proliferation of tissues in culture, although advances in genomic technologies are beginning to generate important clues [18].

The aim of this study was to investigate how shoot explants of adult radiata pines (*P*. *radiata* D. Don) would respond to SE induction on media with varying quantities of 2,4-dichlorophenoxyacetic acid (2,4-D), naphthaleneacetic acid (NAA) or picloram. Comparing tissues induced from primordial shoots with those induced from axillary shoots was another major aspect. In order to provide additional insights into the developmental character of the induced tissues, gene expression profiling was conducted using real-time qPCR. Specifically, transcriptional factors whose expression is reflective of tissue identity were targeted, along with genes linked to cellular metabolism, mitotic and meristematic activity. In order to provide a foundation upon which to compare the primordial and axillary shoot-derived tissues, three EM lines induced from immature zygotic embryos were also analyzed.

## Results and Discussion

### Experimental approach

**Fig 1:**
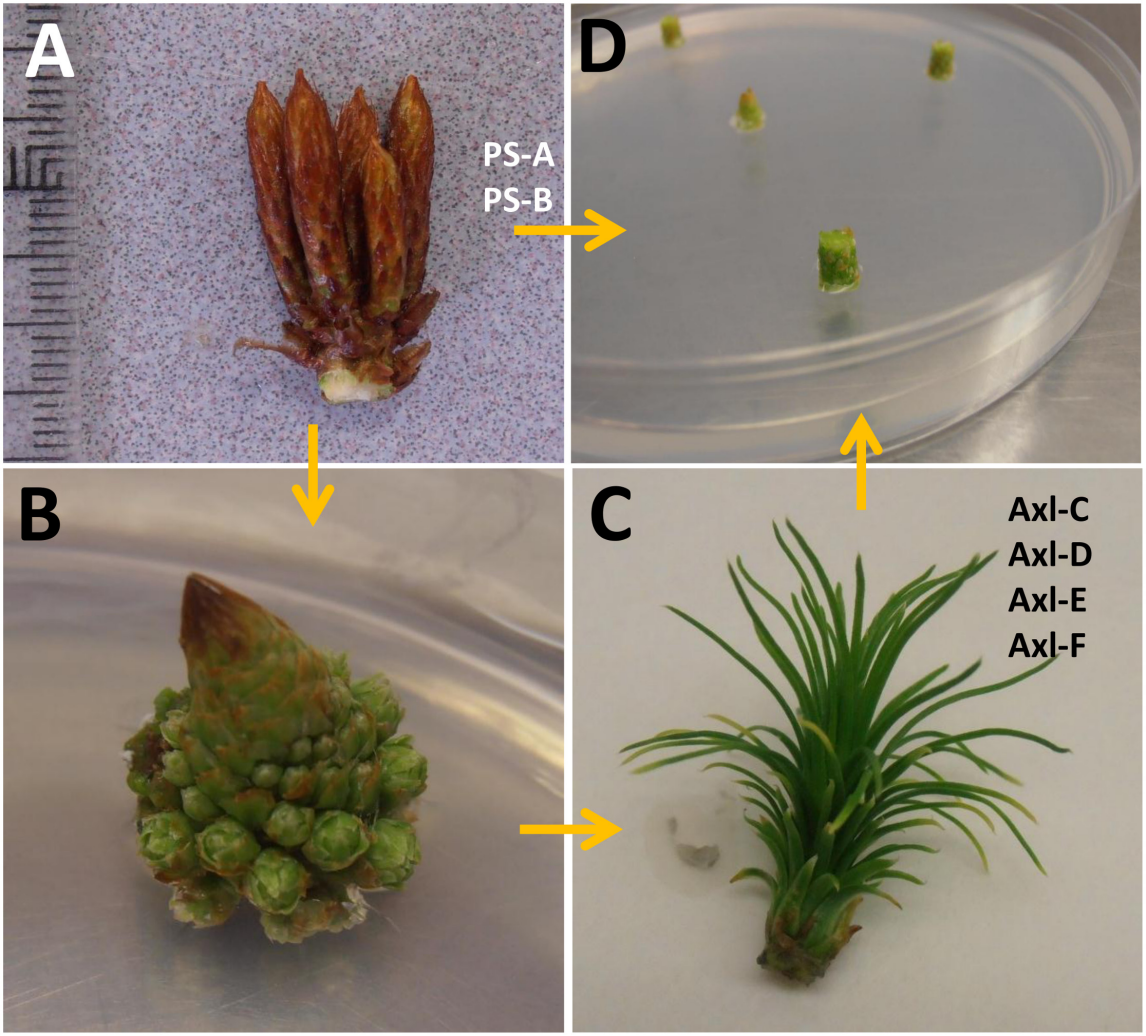
Vegetative explants collected from adult radiata pine trees for culture initiation. A) Pre-flush shoot buds were collected from either adult somatic embryo-derived trees (PS-A and PS-B) or from four adult seed-derived trees. B) Apical sections of primordial shoots collected from the four seed-derived trees were cultured on LP1 medium containing 22 μM BA to promote axillary shoot development. Note the developing needle fascicles (7x). C) PGR-free LP1 medium was then used to promote elongation of axillary shoots, from which 2–3 mm slices were taken for SE induction (7x). D) Embryo development medium (EDM) containing four different PGR compositions (see Materials and Methods) was used for induction of tissues within sections from either primordial shoots collected from two genotypes of somatic embryo-derived trees (PS-A and PS-B), or axillary shoots developed in culture collected from four genotypes of seed-derived trees (Axl-C, Axl-D, Axl-E and Axl-F).

The primary objective of this study was to determine whether SE induction could be achieved within shoot explants collected from adult radiata pine trees, based on three central parameters: genotype, explant type, and auxin composition. This also included shoot explants taken from two somatic embryo-derived trees, based on the premise that they could have greater propensity to undergo SE induction than shoot explants of seed-derived trees. For example, young needles collected from 1-year-old somatic embryo-derived Norway spruce trees displayed a higher SE induction response as compared with explants taken from seed-derived trees of similar age [12]. Furthermore, shoot explants taken from somatic embryo-derived white spruce trees as old as 10 years were shown to be responsive to SE induction [14,15]. It has also been postulated that exposure to PGR in culture increases DNA methylation levels, which in turn may stimulate cell division and differentiation leading to increased propensity for organogenesis or SE induction [13]. Thus, in addition to testing the ability of 2,4-D and 6-benzyladenine (BA) to promote SE induction, as has been reported for zygotic embryos of radiata pine and for shoot explants of white spruce [14], it was of interest to test whether other auxins (NAA and picloram) would be successful in promoting SE induction. Although this approach failed to generate embryonal masses, six callus lines were selected for analysis: two derived from shoot buds of somatic embryo-derived trees and four derived from axillary shoots generated *in vitro* from shoots collected from four seed-derived trees (Fig 1).

### Shoot explant responses

#### Tissue morphology and impact of PGR composition

**Fig 2:**
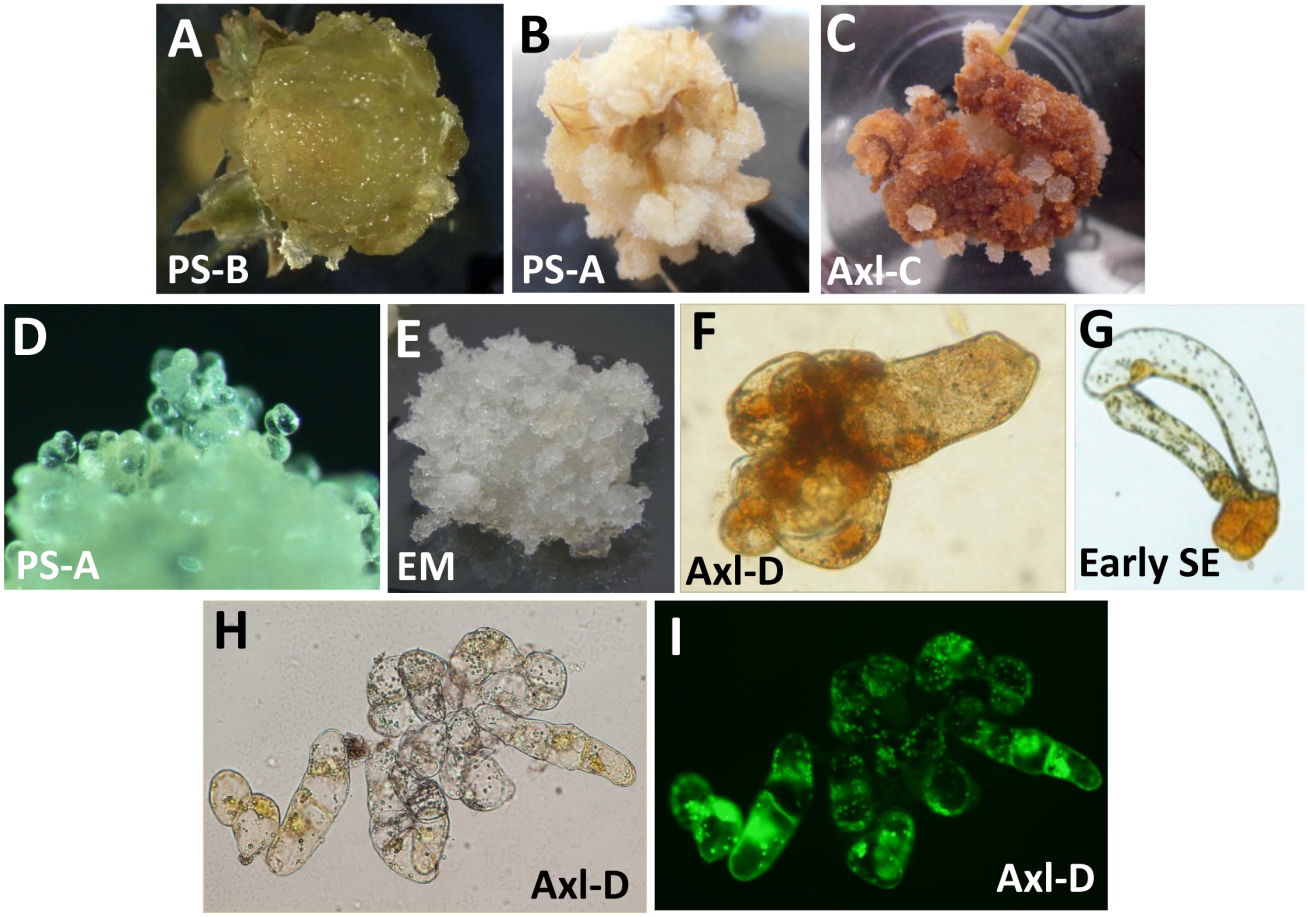
Morphology of tissues induced from primordial and axillary-shoot explants of radiata pine. A) PS-B explant cultured on EDM Pic for 6 weeks (5x). B) PS-A apical shoot explant cultured on EDM-NAA for 6 weeks (4x). C) Axl-C axillary shoot explant cultured on EDM-Pic for 9 weeks (4x). D) PS-A callus composed of loosely associated round cells growing on EDM-4 (100x). E) EM induced from immature zygotic embryos (5x). F) Axl-D cell aggregate stained with KI (400x). G) EM-A early stage somatic embryo stained with KI (400x). H) Bright-field and I) fluorescence microphotographs of an Axl-D cell aggregate stained with fluorescin diacetate (200x).

Explants of all genotypes produced calli of various morphologies, but no predominant characteristic could be attributed to any specific PGR composition. Tissue morphologies ranged from white, translucent and watery in appearance (Fig 2A) to white, compact or cotton-like (Fig 2B). For the two somatic embryo-derived genotypes (PS-A and PS-B), both EDM-1 and EDM-4 (4.5 or 18 μM 2,4-D, respectively) produced more abundant calli as compared with EDM-NAA or EDM-Pic. Other than a greater propensity for necrosis, the axillary shoot explants generated similar tissue morphologies (Fig 2C). Calli generally lacked organized structures, instead containing cells that were round and loosely associated with each other (Fig 2D). Despite their translucent appearance, none of the calli had unambiguous morphological characteristics of EM (Fig 2E). However, some aggregates were composed of more or less round cells and elongated cells (2 to 3 times the size of round cells in length) with some degree of resemblance to early somatic embryos. KI staining revealed the presence of starch grains within these aggregates (Fig 2F), which was also common in other callus lines and within early stage somatic embryos (Fig 2G). Other aggregates contained groups of highly viable cells that were round and elongated and divided in a random fashion that lacked any substantive organization (Fig 2H and 2I).

#### Explant position effect (apical versus basal)

Overall, explants from apical and adjacent sub-apical positions of both primordial and axillary shoots produced more abundant calli than the explants cut from lower positions. However, no discernible differences were observed in the morphology of calli produced from explants taken from apical versus basal positions.

#### Genotype effect and culture age

Calli of genotypes Axl-C and Axl-D started necrotizing in culture sooner than those of genotypes Axl-E and Axl-F. The latter two genotypes supported the growth of white friable cell masses for 12 weeks. After 8 to 10 weeks of culture, growth was slower and parts of the calli became yellow-brown. Calli of genotypes Axl-C and Axl-D became predominantly necrotic with small, round islands of growing white tissue (Fig 2C).

Genotype-dependent response to SE induction within primordial shoot explants of adult conifers has previously been reported for *P*. *contorta*, although only two of fifteen genotypes produced embryogenic-like tissues [19]. Similar to this study, microscopic examination showed both similarities and differences with organized structures typically found within embryogenic cultures. Despite the presence of embryogenic-like structures, none of the induced lines were able to generate mature somatic embryos. Nevertheless, qPCR analysis did reveal expression of the putative embryogenic marker LEC1, indicating that expression profiling has the potential to reveal differences that are not evident from morphological examination.

### Expression profiling of embryonal masses

In order to provide additional insights into the developmental character of the induced tissues, gene expression profiling was conducted using real-time qPCR with two broad objectives. The first was to implement absolute quantification using a method called LRE qPCR, which abrogates the need to construct standard curves [15,20–23]. As detailed below, quantifying gene expression as the number of mRNA transcripts provides a substantive improvement over relative quantification, which is commonly used for expression profiling.

The second objective was to target transcriptional factors whose expression is reflective of tissue identity, with the specific intent to assess the developmental “character” of the induced tissues. Genes linked to cellular metabolism were also analyzed, in part to assess the impact of different PGR compositions within the induction media. In order to provide a foundation upon which to compare the primordial and axillary shoot-derived tissues, three EM lines induced from immature zygotic embryos were also analyzed, which further provides an opportunity to describe each of the five functional categories of genes selected for the analyses.

#### Reference gene

Historically, analysis of genes with a high level of expression stability (generically referred to as “reference genes”) have played a central role in gene expression profiling [24,25]. While the selection and application of reference genes can be complex, reference gene analysis was used in this study to first evaluate the technical variance associated with RNA and cDNA preparation, and then to assess the level of biological variability, with the primary goal of establishing a foundation from which to interpret differences in gene expression.

**Fig 3:**
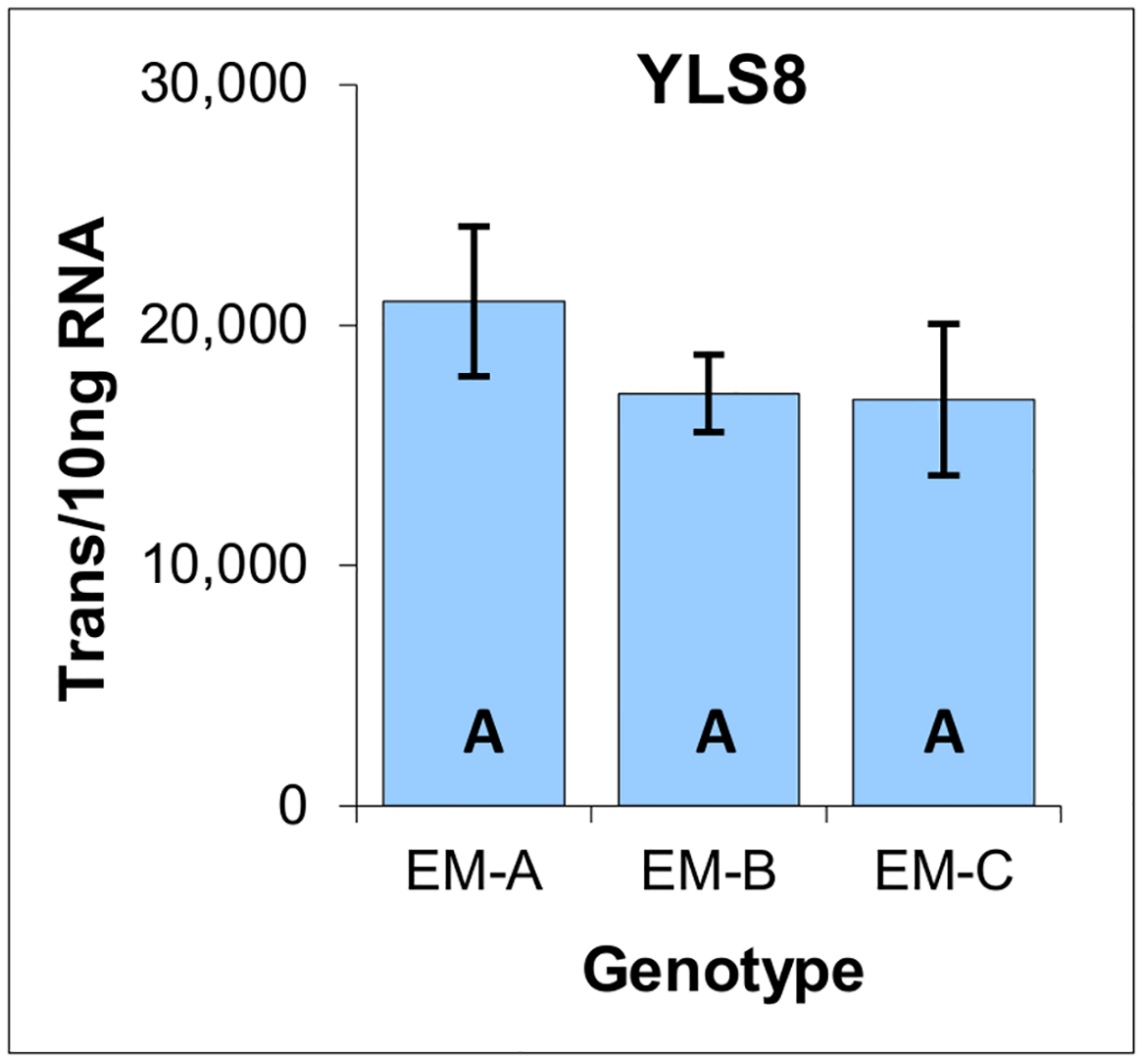
YLS8 reference gene expression within three genotypes of EM induced from immature zygotic embryos of radiata pine. Average of three biological replicates with the standard deviation presented as bars, expressed as the number of YLS8 transcripts per 10 ng of total RNA. Lettering designates no significant differences based on one-way ANOVA analysis (S1 File).

Based on an extensive survey of reference genes conducted during a study of white spruce SE induction from primordial shoots of white spruce [15], YLS8 was selected as a potentially effective reference gene for radiata pine. Indeed, analysis of YLS8 expression within the three genotypes of EM revealed small differences within both biological replicates (intra-genotype: primarily reflective of technical variance) and between the average generated by each genotype (inter-genotype: primarily reflective of biological variability). In addition, one-way ANOVA analysis failed to distinguish any significant differences between the average expression levels generated by the three genotypes of EM (Fig 3).

One of the most significant outcomes of this analysis is the ability of absolute quantification to generate data that is intuitive, and to which basic statistical analysis can be applied easily. For example, the average coefficient of variation generated by the biological replicates (±14.3%) is indicative of a low level of technical variance, and is similar to that reported previously for LRE qPCR-based expression analysis [15,20,22,23]. Similarly, small differences in the average level of YLS8 expression across these three genotypes (±12.5%) are indicative of a low level of biological variability.

Taken together, these data support the ability to directly compare inter-genotype expression data, based on the presumption that differences in expression levels are not due to some form of systemic bias generated, for example, by differences in metabolic activity. Indeed, the fact that YLS8 is a mRNA processing factor suggests that its expression could be broadly reflective of metabolic activity, albeit with limited scope. Nevertheless, the modest differences in YLS8 expression does support direct comparison of transcript quantities across these three EM genotypes without some form of reference gene normalization that is typically used to compensate for technical and biological variance [24,26,27].

#### Mitotic activity

**Fig 4:**
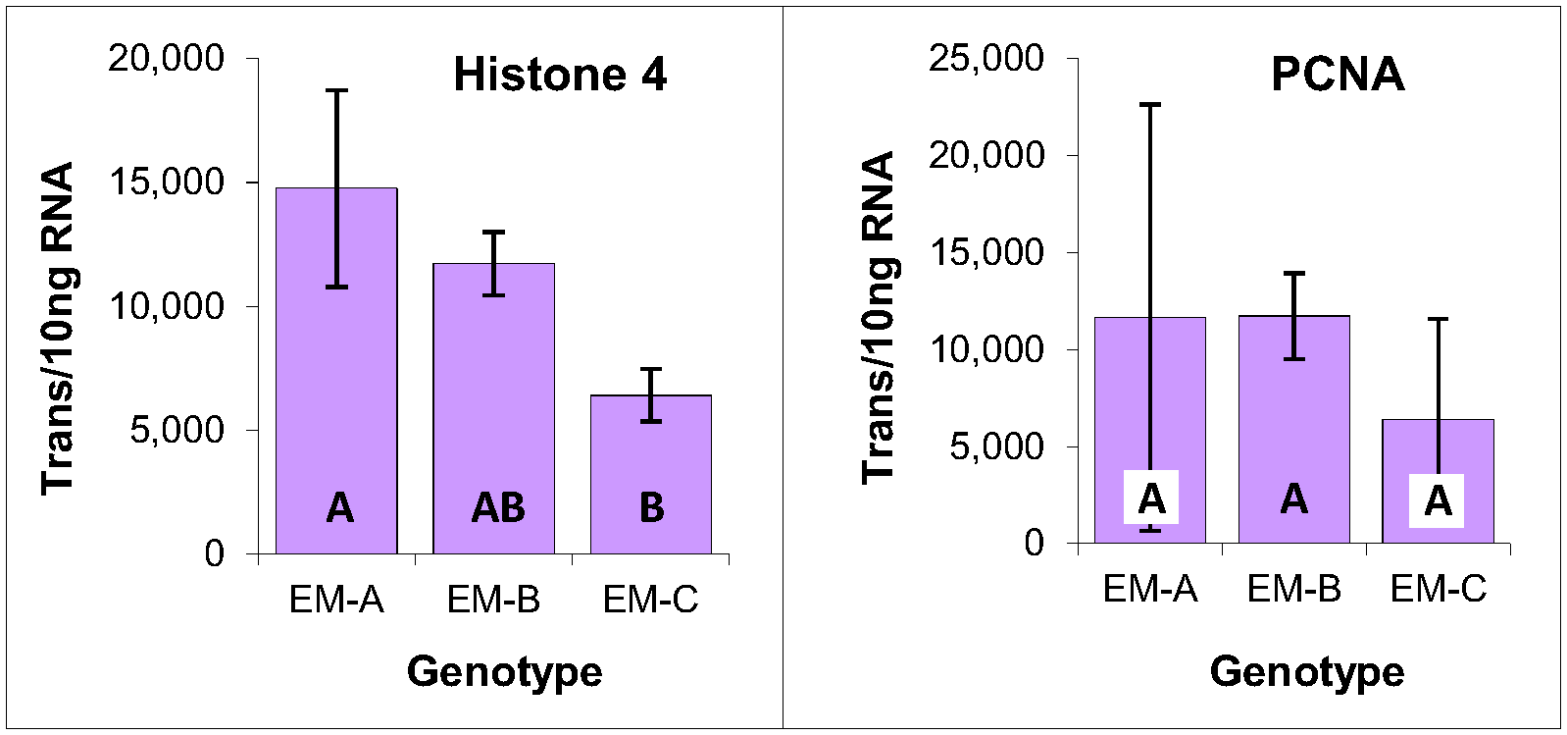
Cell division gene expression within three genotypes of EM induced from immature zygotic embryos of radiata pine. Average of three biological replicates expressed as the number of transcripts per 10 ng of total RNA, with standard deviations presented as bars. Lettering designates significant differences in histone 4 expression based on one-way ANOVA analysis with post-hoc Tukey HSD (p<0.05) (S1 File).

Due to the central role that cell proliferation plays in tissue culture, it was of interest to examine the mitotic activity within each of the EM genotypes. This was accomplished by analyzing the expression of two genes that are directly linked to cell division: histone 4, a component of the nucleosome around which DNA is coiled to form chromatin, and PCNA, a DNA polymerase processivity factor, both of which are expressed exclusively during S-phase. Analysis of their expression revealed moderate differences, suggesting that genotype EM-C has a mitotic rate that is about 50% lower than that of the other genotypes (Fig 4).

A somewhat similar result was reported for Norway spruce in which differences in growth rate within two EM lines generated a difference a little over 0.5-fold in the apparent expression level of WOX2, which is a marker of embryogenic character [28]. Nevertheless, such relatively small differences in mitotic rate are expected to impact absolute transcript quantities only modestly, if at all, as was found for WOX2 expression in these three radiata pine EM genotypes (see below).

#### Embryogenic markers

One of the primary objectives for expression profiling was to assess the embryogenic character of the shoot-derived tissues. Conifer homologs of three embryo-specific angiosperm genes were thus selected for analysis (LEC1, WOX2 and ABI3) based in part on reports that their expression are potential markers of embryogenic character in conifers [14,19,28–30].

LEAFY COTYLEDON1 (LEC1) is a novel NF-YB subunit of the CCAAT-binding transcription factor that functions as a master regulator of embryogenesis [31–33]. WOX2 is a member of the Wuschel-related homeobox gene family [34] whose expression is associated with specifying apical cell identity during the earliest stages of Arabidopsis embryogenesis [35]. ABSCISIC ACID INSENSITIVE3 (ABI3) is a major regulator of seed maturation in Arabidopsis and is a member of the B3-domain family of transcriptional factors [36].

**Fig 5:**
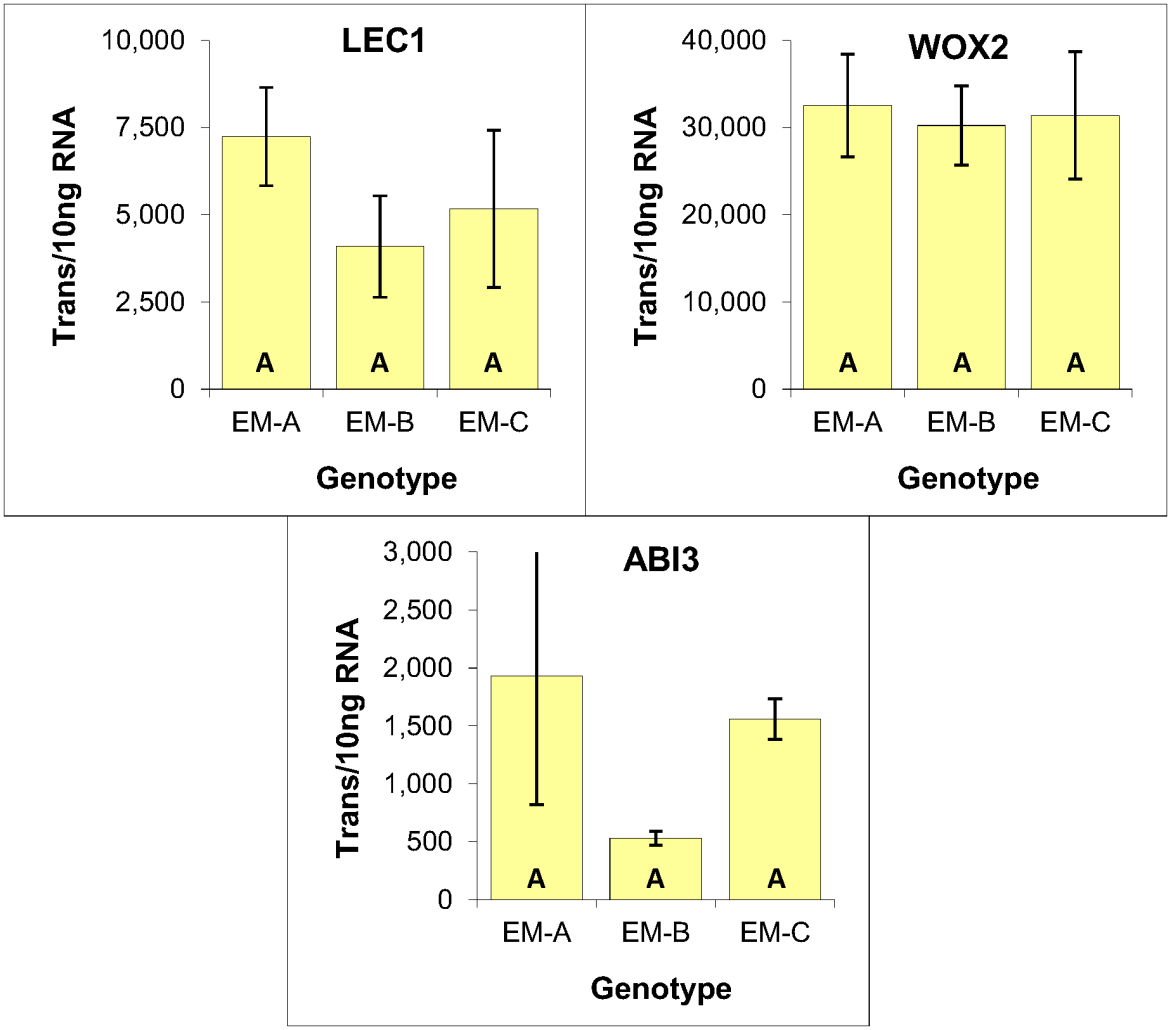
Expression of embryogenic markers within three genotypes of EM induced from immature zygotic embryos of radiata pine. Average of three biological replicates expressed as the number of transcripts per 10 ng of total RNA, with standard deviations presented as bars. Lettering designates no significant differences based on one-way ANOVA analysis with post-hoc Tukey HSD (p<0.05) (S1 File).

Expression analysis of LEC1, WOX2 and ABI3 revealed no significant differences based on one-way ANOVA analysis. These results thus provide no indication that the lower mitotic rate of genotype EM-C impacted the expression level of any of these three genes, further illustrating the similarity of these three genotypes (Fig 5).

#### KNOX meristematic markers

Conifers possess four genes encoding for KNOX1-like genes (SKN1-4) [37], which have been found to play a pivotal role in meristem cell maintenance and identity within angiosperms [37,38]. Previous analysis of the Norway spruce KNOX gene expression during somatic embryo maturation indicated that the closely related SKN1 and SKN2 genes (referred to as HBK3 and 1, respectively) have a generalized function, whereas SKN3 and SKN4 expression (referred to as HBK2 and HBK4 respectively) is correlated with the formation of the shoot apical meristem, although these two genes are also expressed in EM [38,39].

**Fig 6:**
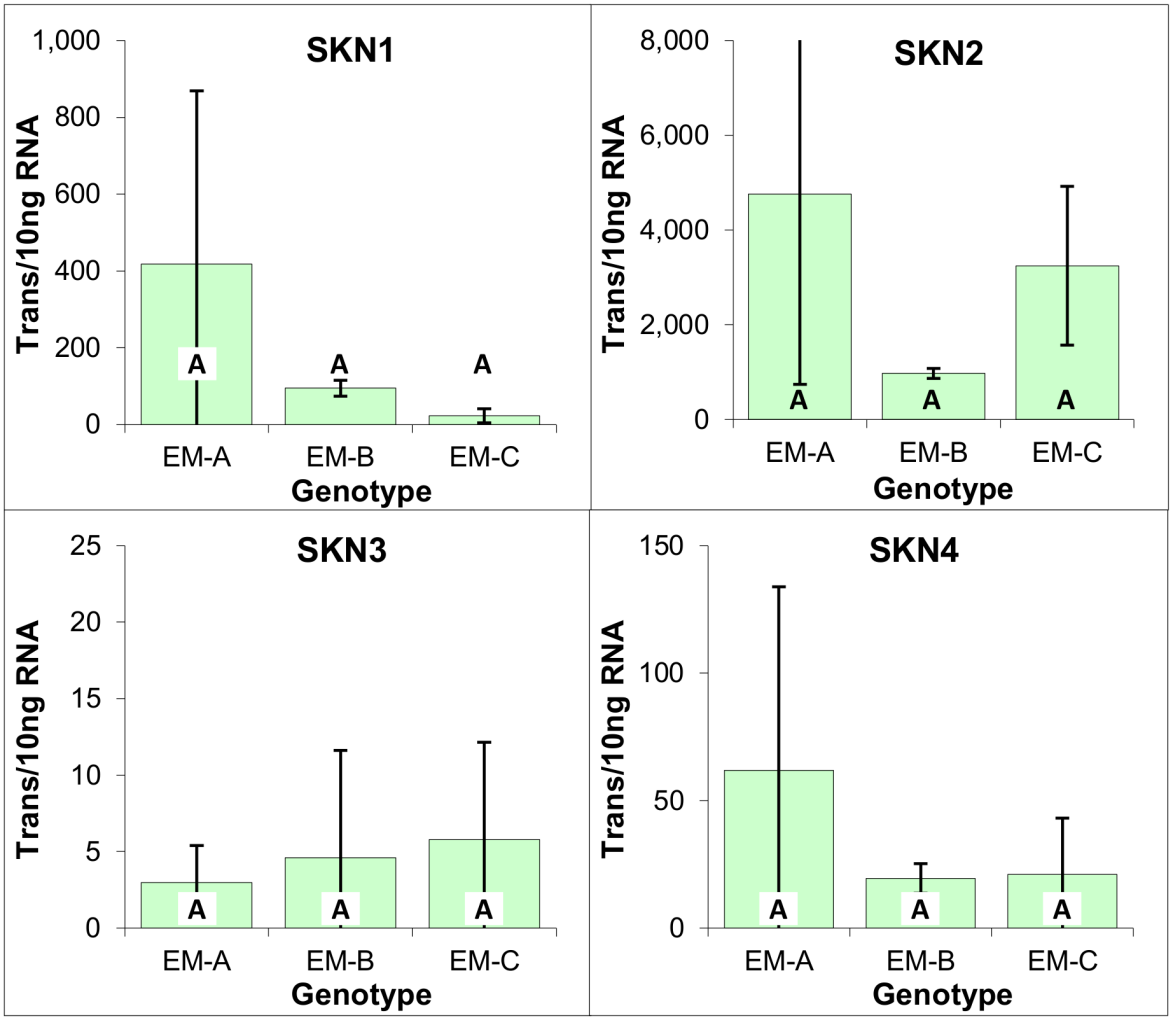
Expression of meristematic markers within three genotypes of EM induced from immature zygotic embryos of radiata pine. Average of three biological replicates expressed as the number of transcripts per 10 ng of total RNA, with standard deviations presented as bars. Lettering designates no significant differences based on one-way ANOVA analysis with post-hoc Tukey HSD (p<0.05) (S1 File).

Similar to what has previously been reported for white spruce EM [14], analysis of the three radiata pine EM genotypes revealed that SKN1 and SKN2 were the most predominantly expressed, with SKN2 expression being over 10 times higher on average than that of SKN1. Furthermore, expression of SKN3 and SKN4 was apparent, albeit at a much lower level, with SKN3 expression being just detectable (Fig 6).

#### Vegetative marker WOX4

**Fig 7:**
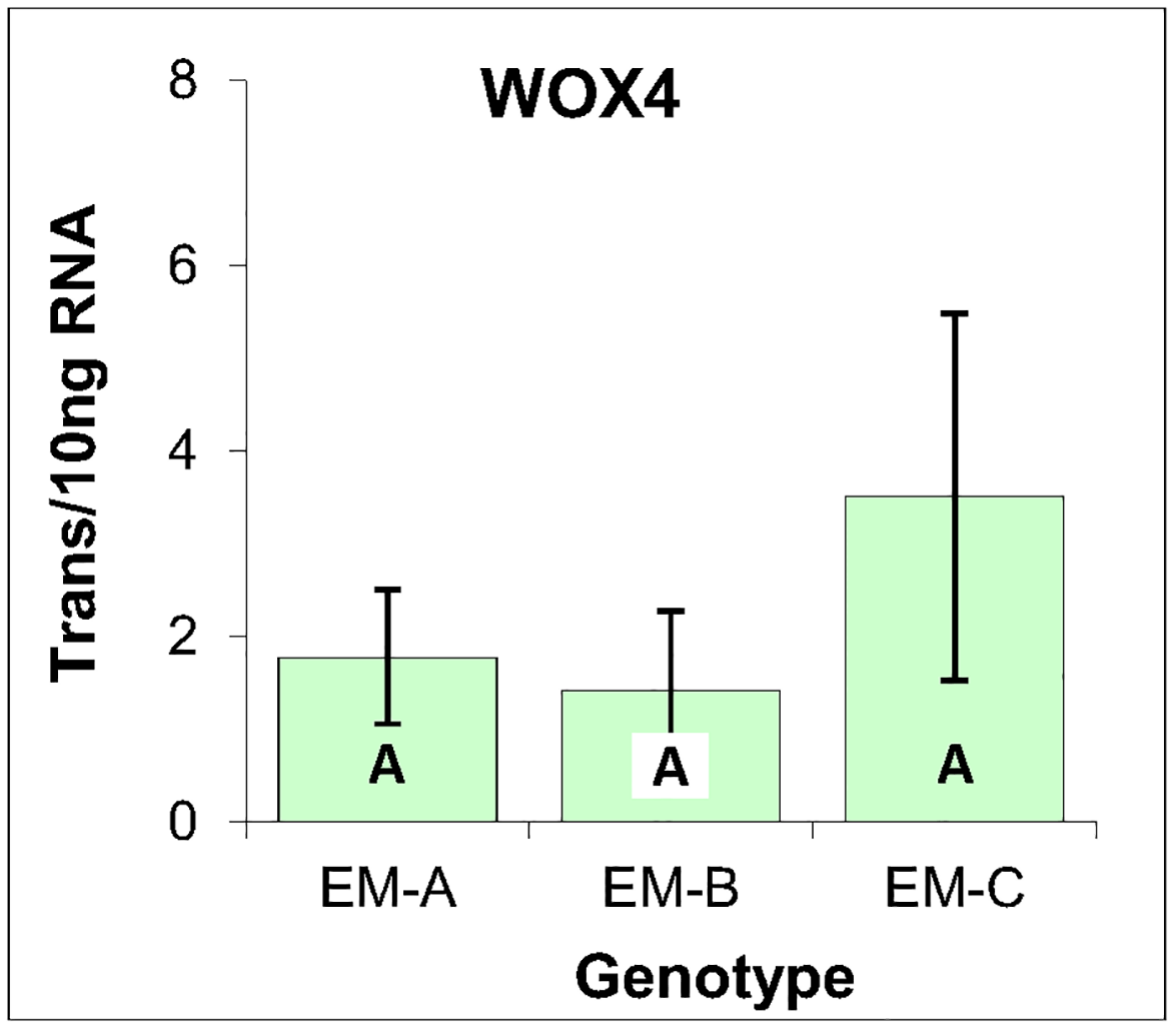
WOX4 gene expression markers within three genotypes of EM induced from immature zygotic embryos of radiata pine. Average of three biological replicates expressed as the number of transcripts per 10 ng of total RNA, with standard deviations presented as bars. Lettering designates no significant differences based on one-way ANOVA analysis with post-hoc Tukey HSD (p<0.05) (S1 File).

To expand the scope of the analysis and include a marker of vegetative tissues, the expression of WOX4 was analyzed. Based on work conducted in angiosperms, WOX4 expression is associated with vascular procambium tissue [40,41], as has been confirmed for the Norway spruce homology, in that it is maximally expressed within cambium and shoot tips [42]. Indeed, the ultra-low level of WOX4 expression within the three EM genotypes is consistent with the unvascularized nature of conifer EM (Fig 7).

### Expression profiling of primordial and axillary shoot-derived calli

Particularly in view of similarities in culture morphology, it was of interest to determine whether differences in gene expression patterns were associated with PGR composition and/or explant type. Note that genotype Axl-F was excluded from the qPCR analysis due to poor tissue condition. A single biological replicate was collected from tissues grown on each of the four different PGR compositions and the number of mRNA transcripts per 10 ng of total RNA determined. MS Excel two-way ANOVA without replication failed to reveal significance differences in gene expression across the four media types, indicating that PGR composition had no discernible impact on gene expression (S2 File). Transcript quantities for each genotype-target combination were thus averaged across the four media.

#### Reference gene

**Fig 8:**
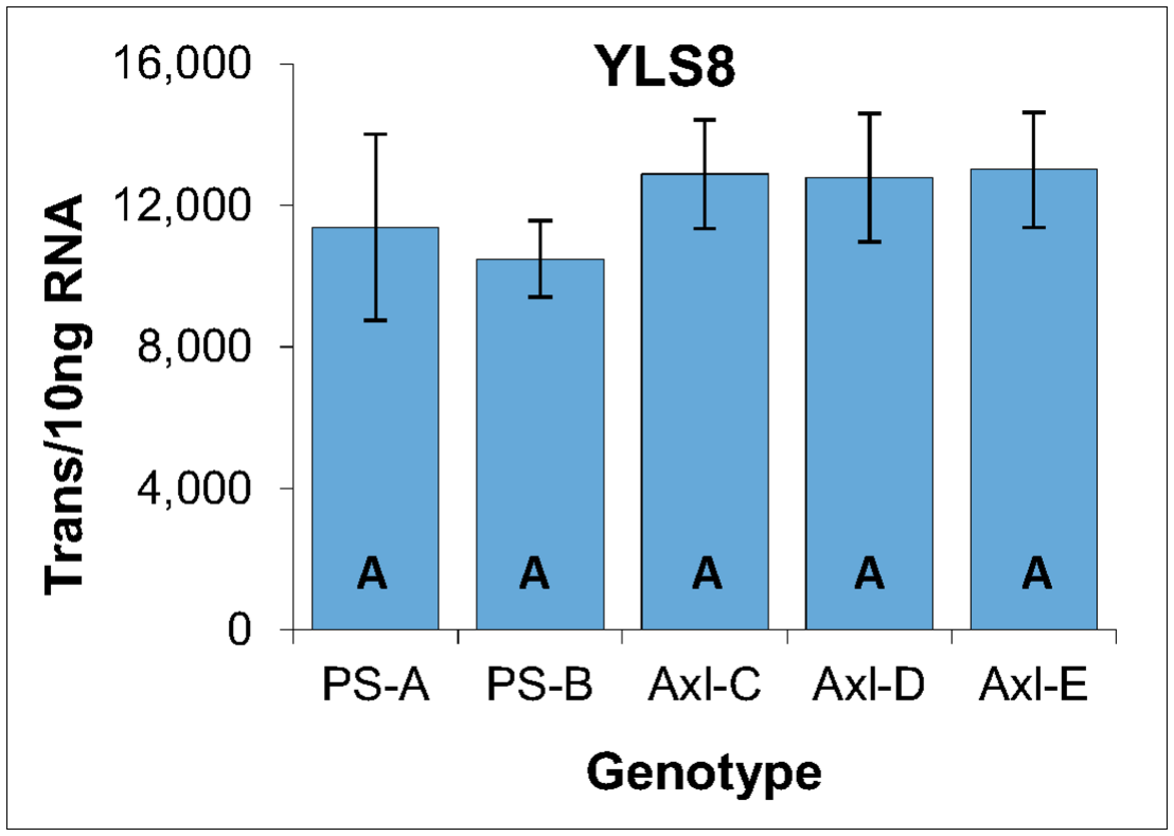
YLS8 expression within primordial and axillary shoot-derived tissues of radiata pine. Expressed as the number of YLS8 transcripts per 10 ng of total RNA, with standard deviations presented as bars. Lettering designates no significant differences based on one-way ANOVA analysis with post-hoc Tukey HSD (p<0.05) (S3 File).

As was observed in the three EM genotypes (Fig 3), similarity in the YLS8 expression is indicative of low levels of both technical and biological variance, as well as of a broad similarity in metabolic activity, as based on the role of YLS8 in RNA processing (Fig 8).

#### Mitotic activity

**Fig 9:**
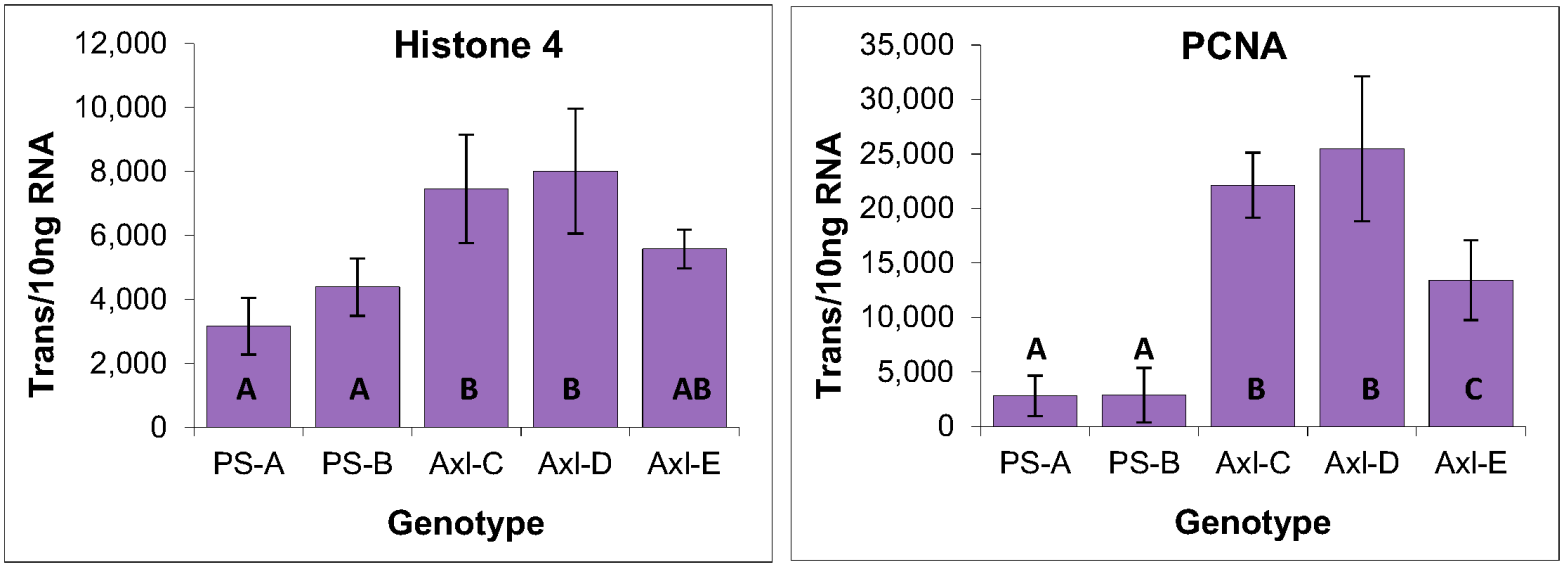
Histone 4 and PCNA expression within primordial and axillary shoot-derived tissues of radiata pine. Expressed as the number of YLS8 transcripts per 10 ng of total RNA, with standard deviations presented as bars. Lettering designates significant differences based on one-way ANOVA analysis with post-hoc Tukey HSD (p<0.05) (S3 File).

Low levels of PCNA expression within the two primordial shoot-derived lines is indicative of lower mitotic activities, which is consistent with the lower levels of histone 4 expression as compared with the three axillary shoot-derived lines. Note also that Axl-E generated an intermediate level of expression for both histone 4 and PCNA as compared with the other four genotypes (Fig 9).

#### Expression of embryogenic and meristematic markers

**Fig 10:**
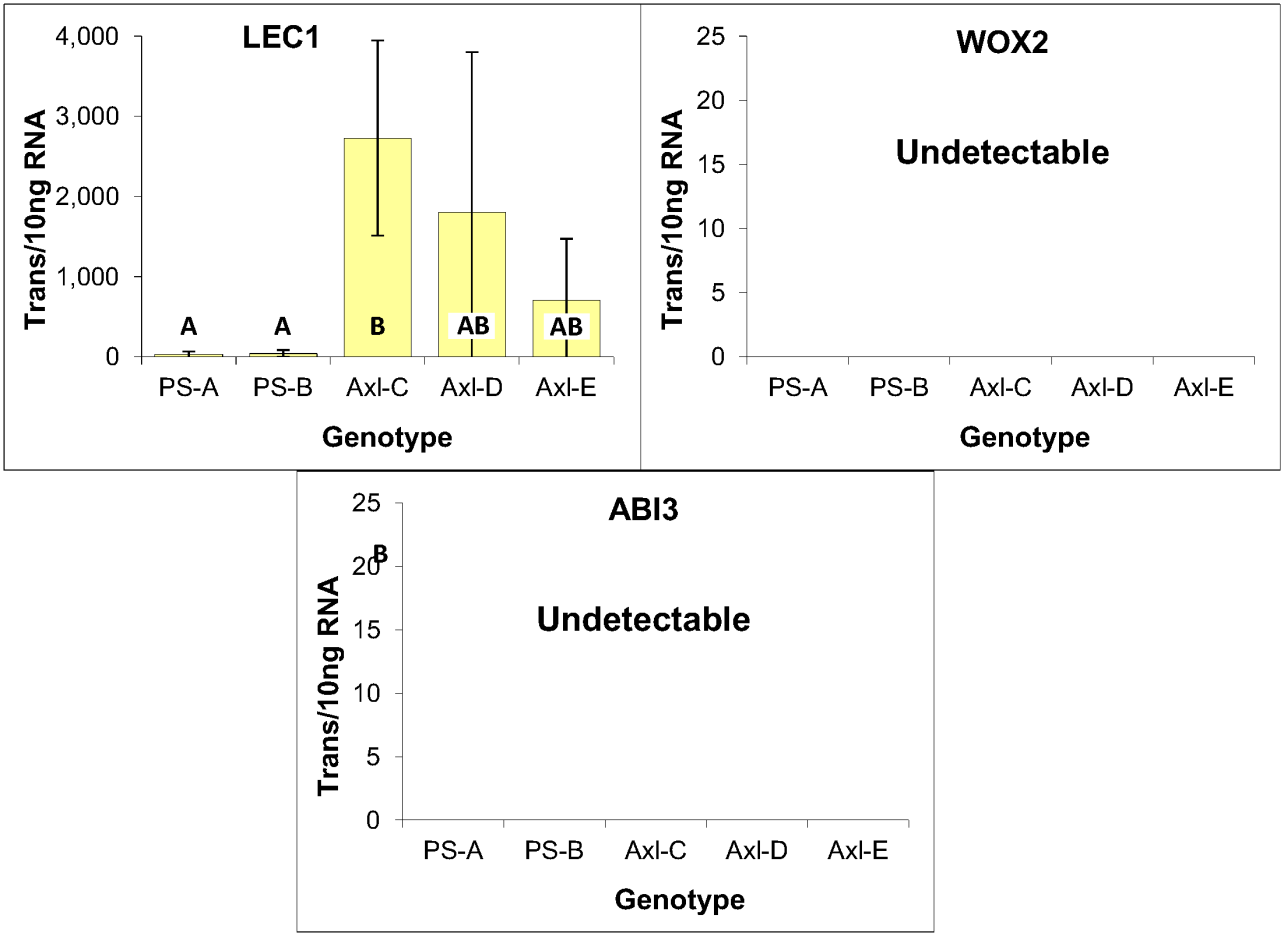
Embryogenic marker expression within primordial- and axillary-shoot derived tissues of radiata pine. Expressed as the number of mRNA transcripts per 10 ng of total RNA, with standard deviations presented as bars. Lettering designates significant differences based on one-way ANOVA analysis with post-hoc Tukey HSD (p<0.05) (S3 File). Note that the level of LEC1 expression within the three axillary shoot-derived genotypes is comparable to that seen in EM (Fig 5), albeit of greater variability.

While expression of WOX2 and ABI3 were undetectable, LEC1 was found to be expressed within all five callus genotypes with the three axillary shoot-derived lines generating levels comparable with those observed in EM (Fig 10). Although the absence of embryo-like structures within these cultures is consistent with the lack of WOX2 and ABI3 expression, the biological significance of LEC1 expression is unclear. This is further compounded by the apparent explant-specific differences in LEC1 expression, with the three axillary shoot-derived lines expressing LEC1 48 times more on average than the two primordial shoot-derived lines (Fig 10). Although the lower mitotic activities of the primordial shoot-derived lines likely contributed to the lower transcript levels, such a bias is small in relation to the magnitude of these differences.

Importantly, LEC1 expression has also been reported within calli induced from shoot explants of lodgepole pine (*P*. *contorta* Dougl. ex Loud) [19]. Furthermore, overexpression of a conifer LEC1 gene (CHAP3A) within transgenic somatic seedlings of white spruce was previously found to have no impact on either seedling morphology or expression of ten other embryo-related genes [29]. Thus, although it is not possible to completely exclude that LEC1 expression within callus and vegetative tissues has no biological implications, these data do suggest that LEC1 expression alone, even at relatively high levels, is insufficient to generate any apparent embryonic character, possibly due to a lack of other somatic embryo-related factors.

**Fig 11:**
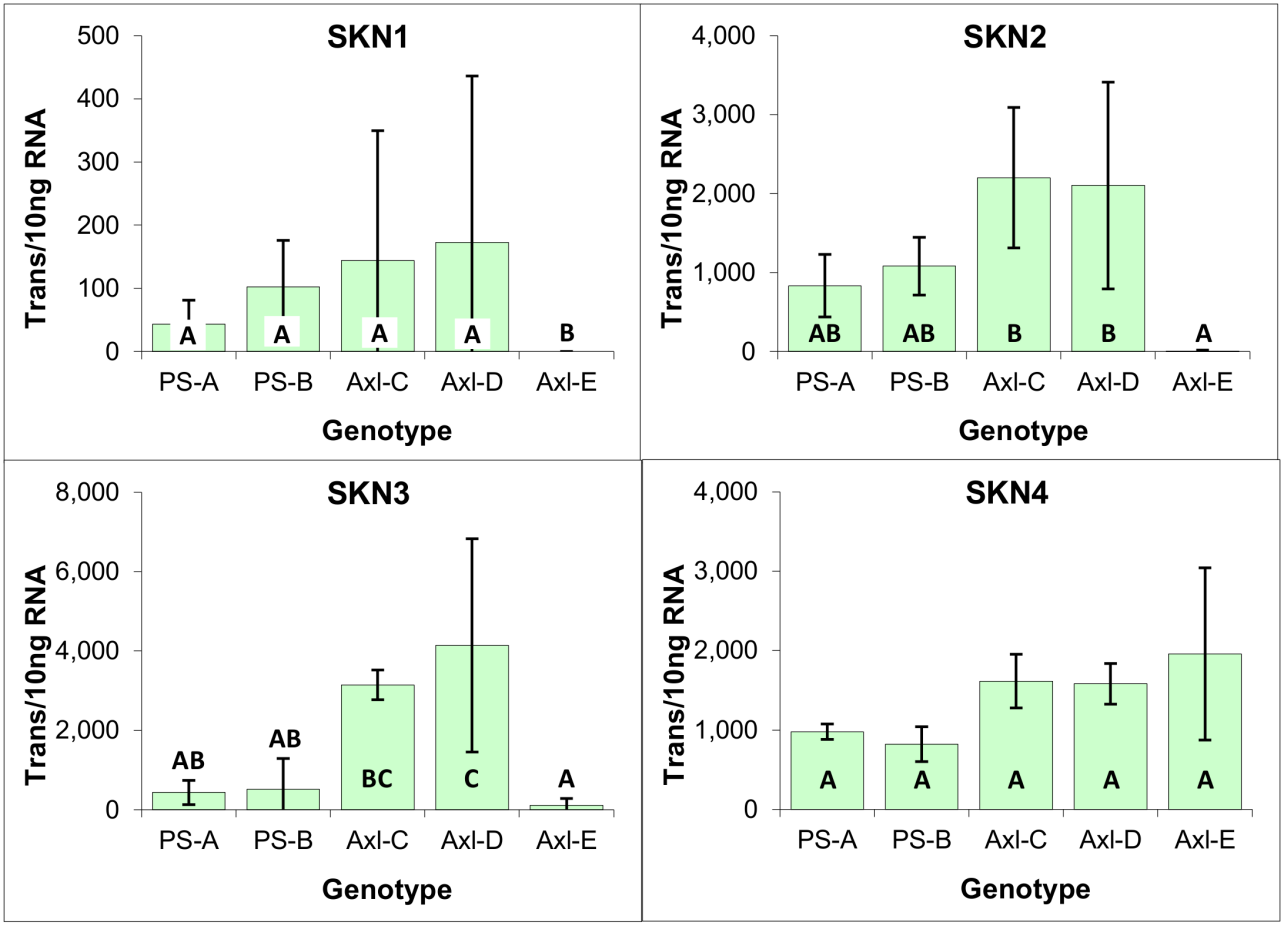
Meristematic marker expression within cultures derived from primordial and axillary shoot explants of radiata pine. Expressed as the number of mRNA transcripts per 10 ng of total RNA, with standard deviations presented as bars. Lettering designates significant differences based on one-way ANOVA analysis with post-hoc Tukey HSD (p<0.05) (S3 File).

With the exception of Axl-E, all genotypes expressed SKN1 and SKN2 at levels similar to those observed in EM, supportive of a generalized function; however, high level expression of SKN3 and SKN4 is consistent with the vegetative origin of these calli lines, in that expression of those genes has been associated with shoot apical meristems [42] (Fig 11). The Axl-E genotype was exceptional in that although it expressed SKN4 at levels comparable with those of the other lines, expression of the other three SKN genes was close to undetectable. The biological significance of this exclusive expression of SKN4 is unknown, but it does suggest some fundamental difference in the developmental character of this callus line.

**Fig 12:**
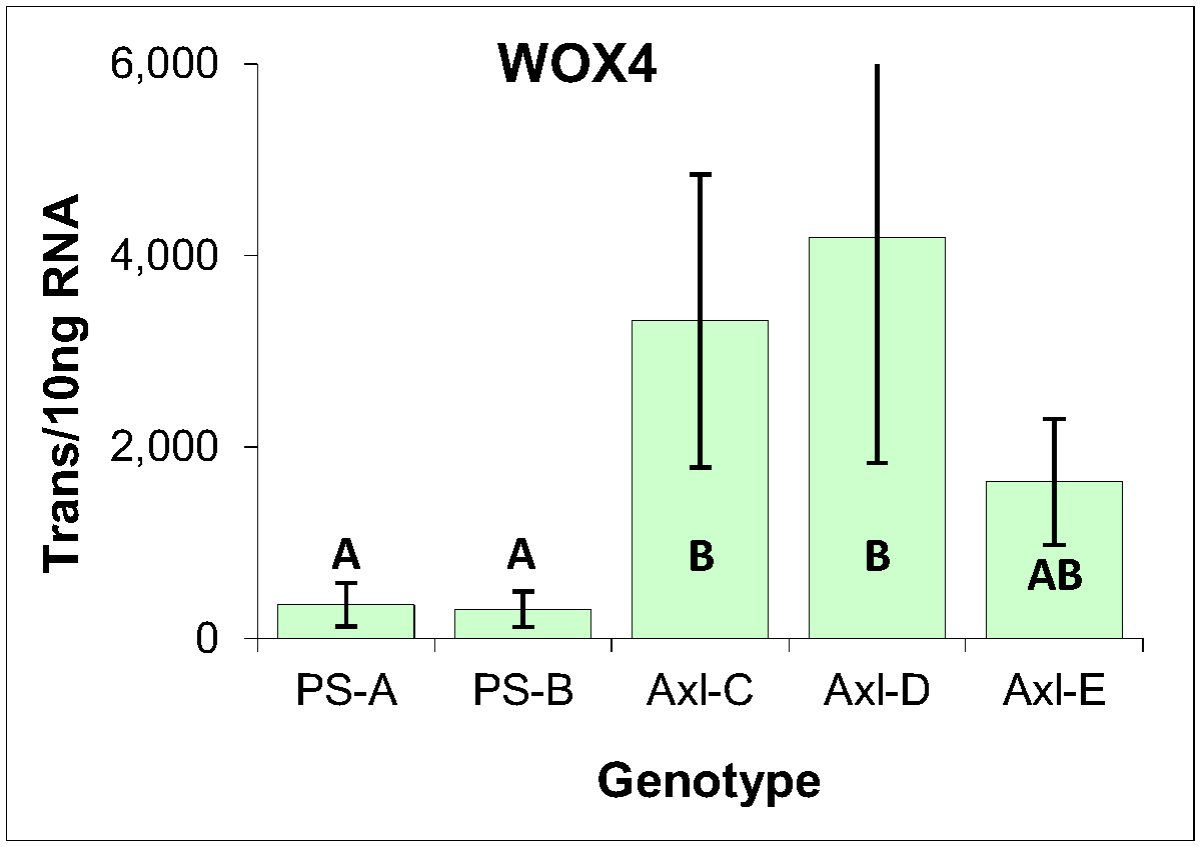
WOX4 gene expression within cultures derived from primordial- and axillary-shoot explants of radiata pine. Expressed as the number of mRNA transcripts per 10 ng of total RNA, with standard deviations presented as bars. Lettering designates significant differences based on one-way ANOVA analysis with post-hoc Tukey HSD (p<0.05) (S3 File).

Consistent with the expression of apical meristem markers SKN3 and SKN4, the vegetative character of all five callus lines was supported by the expression of vascular procambium marker WOX4. Also similar to what was observed for LEC1, and consistent with the modestly lower mitotic activities of the primordial shoot-derived calli, the axillary shoot-derived genotypes were found on average to express WOX4 at higher levels than the primordial shoot-derived genotypes (Fig 12).

## Conclusions

This study investigated the ability of either primordial shoots or *in vitro* generated axillary shoots to respond to media with four different PGR compositions, including one routinely used for SE induction from zygotic embryos of radiata pine. The explants produced various phenotypes of calli, parts of which were macroscopically reminiscent of embryogenic cultures in texture and color. However, microscopic examination of the five callus lines failed to reveal any EM. Gene expression analysis revealed that induction media had no significant impact, although the two primordial shoot-derived callus lines were found to have a mitotic rate about 50% lower than that of the three axillary shoot-derived lines. High level expression of two Knotted1-like genes further reflected the vegetative character of these callus lines, as was further confirmed by the expression of WOX4, a marker of vascular procambium tissue. Most notable was expression of the embryogenesis gene LEC1 in all five callus lines, although expression of two other embryogenic markers (ABI3 and WOX2) was undetectable. While its biological significance is unclear, LEC1 expression could be reflective of some level of embryogenic character that may have been sequestered by the lack of additional embryonic factors.

## Materials and Methods

### Tissue culture of shoot explants

Note that the collection of field material did not require permission nor did it involve any endangered or protected species.

Pre-flush shoot buds (Fig 1A) from two genotypes (PS-A and PS-B) were collected from 6-year-old radiata pine somatic trees growing in Arkaute (Álava) (latitude: 42°51'N, longitude: 2°37'W), wrapped in moist paper to prevent dehydration, and stored in polyethylene bags at 4°C for a maximum of 4 days. For disinfection, buds were rinsed under running water for 5 min followed by submerging in a 50% solution of commercial bleach (active chloride >5%) for 35 min. Afterwards, the buds were rinsed three times in sterile distilled water, each rinse lasting 5 min. Bud scales were then removed and the primordial shoots were cut transversely with a surgical scalpel blade into 4–5 mm slices (Fig 1D). Slices were placed onto four induction media consisting of EDM [43] containing 30 gL^-1^ sucrose, solidified with 3 gL^-1^ of gellan gum (Phytagel, Sigma-Aldrich) and supplemented with BA, 2,4-D, NAA or picloram (Table 1). Every slice was sequentially numbered to depict apical to basal positions.

**Table 1:** Induction medium composition.

	BA	2,4-D	NAA	Picloram
**EDM-1**	2.7 μM	4.5 μM	0	0
**EDM-4**	2.7 μM	18.0 μM	0	0
**EDM-NAA**	2.7 μM	4.5 μM	13.5 μM	0
**EDM-Pic**	2.7 μM	0	0	20 μM

*In vitro* generated axillary shoots of four additional genotypes (Axl-C, Axl-D, Axl-E, and Axl-F) were obtained by following the method described in Montalbán et al. [10]. Note that in part due to the length of time required (>1 year), the production of axillary shoots was conducted as a separate project from that of the somatic trees pre-flush buds described above. Briefly, 1-cm slices of primordial shoots collected from trees approximately 20 years old and located in a seed orchard established by Neiker-Tecnalia in Deba (Guipúzcoa, Spain) (latitude: 43°10’N, longitude: 2°10’W) were placed onto medium based on LP [44] modified by Aitken-Christie and Singh [45], designated as LP1, and supplemented with 22 μM BA. After 30–45 days in culture (when elongating needle fascicles were evident, Fig 1B), the explants were placed onto LP1 medium without BA and supplemented with 2 gL^-1^ activated charcoal; pH was adjusted to 5.8. The axillary shoots were transferred onto fresh medium after 5–7 weeks. Once shoots were approximately 1 cm long, they were separated and cultured individually on fresh medium (Fig 1C). The developing axillary shoots were separated and cut again into sections to promote growth of new axillary shoots. Once a sufficient number of axillary shoots was generated, 2–3 mm slices were cultured on induction media containing different PGR compositions (Table 1).

Eight to ten explants were cultured per 9 x 1.5 cm Petri dish containing 20 ml of medium and cultured in the dark at approximately 23°C. After 6 weeks, the explants were transferred onto fresh media of the same composition and cultured for an additional 6 weeks before final observations and callus sampling were conducted.

### Induction of embryogenic cultures from immature zygotic embryos

Immature radiata pine cones were collected from open-pollinated trees in a seed orchard established by Neiker-Tecnalia in Deba (Guipúzcoa, Spain) (latitude: 43°10’N, longitude: 2°10’W) in June 2012. EM cultures were induced from developing zygotic embryos as previously described [46,47]. Three EM genotypes (EM-A, EM-B and EM-C), confirmed to generate somatic embryos using a standard maturation protocol [47], were selected for gene expression analysis.

### Morphological characterization

Calli of different origin and grown on different culture media (Table 1) were compared macro- and microscopically utilizing either 1% potassium iodide (PI) to stain starch or 0.02% fluorescin diacetate to determine cell viability. After staining, the cell preparations were viewed using bright-field or fluorescence microscopy.

### Gene expression profiling using absolute real-time qPCR

Eleven genes were selected for expression profiling with the primary objective of distinguishing differences in tissue composition across the induced calli and for comparison with zygotic embryo-derived cultures known to be embryogenic based on their ability to produce somatic embryos. The strategy taken was to use a combination of transcriptional factors known to be markers of embryonic (LEC1, ABI3, WOX2), meristematic (WOX4, SKN1, SKN2, SKN3 and SKN4), or mitotic activity (histone 4 and PCNA), in addition to a marker of general metabolic activity (YLS8) that also served as internal quality controls for assessing the technical variance associated with sample preparation and LRE qPCR analysis, as previously reported [15].

Primers were selected from an extensive collection previously used to analyze white spruce, and designed as described previously [15]. Primers were compared to sequence alignments generated from pine EST sequences retrieved from GenBank, and new primers were designed to include pine-specific polymorphic bases when necessary. To verify the absence of polymorphic sequences that significantly disrupt primer annealing and elongation, primer pairs were validated via amplification of gDNA using identical conditions to that used for cDNA amplifications (S4 File). Table 2 lists the primer pairs used in this study, along with the average amplification efficiencies generated by each primer pair.

**Table 2:** List of qPCR primers.

Target	Accession	5’	3’	Av. Emax*
**YLS8**	Psi.7532	CCCGTCTACTGTCATGTTTTTCTTCCGC	CTTGGGGGCAATGACCAGACC	100.7% ±0.7%
**Histone 4**	CX652935	GAAGAGGCAGGGAAGGACTCTCTATGG	ACTGACAGAGAAAGCAACATTGCATACC	101.1% ±0.6%
**PCNA**	DR020478	CATGAACTCGTTCACCAAGGCAACTCC	TTAGCTTTCACCTTCCTCCTCAATCTTAGG	100.2% ±0.7%
**LEC1**	Pgl.10241	CAAGTTGGGTTTTGATGATTATGTGGAGCC	CCCATTCCTGAGGTGCCATAAAGAG	100.4% ±0.7%
**WOX2**	DT638160	TTACAACTTCCAGCATTCACATGACAGT	CTACTTGCCAGGATGCTGAGGGAT	100.6% ±0.7%
**ABI3**	Pgl.6812	GGATCTGACGATCGAAGAGATTTTGGACC	CTCCGATATTATCAGGACTTTTGCCTGGG	102.9% ±0.7%
**SKN1**	Pta.9693	GTAATGAACAGTCACAGTCCTCACAGTGCTG	GCATTGTGTGCTGTTTTCAGCCGAAG	100.3% ±0.6%
**SKN2**	DR091039	GGTCAGAGTCCTCACGGTGCCA	GGACGGAAAATTACAGCTTCCCTTAATACCC	100.9% ±0.5%
**SKN3**	CO366662	ACATCTCAGGACAGATGGAACTGCC	TGAAACATGGCCCATTTTAGAGATCCAGC	101.4% ±0.8%
**SKN4**	GT242369	GGTCGCTTTGGCTGAAAGTACTGGTC	GCAAGGCGATCAACCGAACAGACG	100.4% ±0.6%
**WOX4**	FE524554	CCAATATGGACAACTCAGTATTATCCTTGCACG	GACAACAATTTCATGTCATGGAATTCAAGAAGG	100.3% ±0.5%

*Average LRE-derived amplification efficiency (n = 32)

RNA extraction, reverse transcription and real-time qPCR were conducted as described previously [15]. Briefly, RNA was extracted from tissue samples using a Qiagen Tissue Lyser II (Qiagen, Toronto, ON) bead mill for tissue disruption, and RNA was purified using a Qiagen Qiacube DNA/RNA purification robot and Qiagen RNEasy plant mini kit (Cat. # 74904), including a column DNase treatment (Qiagen RNase free DNase, Cat. #79254). RNA was quantified using a Nanodrop 1000 Spectrophotometer (Thermo Scientific, Waltham, MA) and RNA integrity was assessed using the Agilent 2100 Bioanalyzer (Agilent Technologies Santa Clara, CA) according to the manufacturer’s protocol, which generated RIN values of 9–10.

Reverse transcription was conducted in 20 μl reactions containing 50 ng μl^-1^ RNA, 25 ng μl^-1^ oligo dT primer (Invitrogen, Cat. # 18418–012), 50 μM dNTPs (Invitrogen, Cat. # 18427013), and 5 U μl^-1^ Superscript III (Invitrogen, Cat. # 18064–014) using the manufacturer’s supplied buffer. Reactions were incubated at 42°C for 50 min, and 180 μl 10 mM Tris, pH 8.0 was added to obtain the final concentration of 5 ng total RNA per μl. PCR amplification was conducted with an Applied Biosystems 7500 Fast qPCR system (normal ramping) using 96 well BrightWhite plates sealed with MicroAmp film and QuantiTect enzyme formulation using a 10 μl reaction volume containing 500 nM of primers and an aliquot of reverse transcriptase reaction equivalent to 5 ng RNA (1.0 μl). The cycling regime consisted of a 15 min activation at 95°C, followed by 50 cycles of 95°C for 10 s, and 65°C for 120 s. Amplicon T_m_ was determined for each amplification reaction by melt curve analysis (65 to 90°C) conducted at the end of each run, which produced a single prominent amplicon peak for all primer pairs. Raw fluorescence readings were imported into the LRE Analyzer program (Ver 0.9.7) [23], from which absolute quantities of each target (number of transcripts per 10 ng RNA) were determined using linear regression of efficiency (LRE) and optical calibration as previously described [15,20–22].

### Statistical analysis of expression data

For the three EM genotypes three biological replicates were analyzed and the significance of differences in average gene expression levels assessed using pairwise one-way ANOVA using post-hoc Tukey HDS analysis (p<0.05) as implemented by N. Vasavada [48] (S1 File). Due to limiting tissue mass, single biological replicates were analyzed for the shoot-derived lines collected from tissues grown on each of the four induction media. MS Excel two-way ANOVA without replication failed to reveal any significance differences (p<0.05) in gene expression across the four media for any of the genotypes, indicating that induction medium type had no discernible impact on gene expression (S2 File). Expression data for each axillary shoot genotype is thus presented as the average across the four media. Assessment of the significant of inter-genotype differences in gene expression was conducted using pairwise one-way ANOVA analysis using post-hoc Tukey HSD (p<0.05) as implemented by N. Vasavada [48] (S3 File).

## Supporting Information

S1 File Pairwise one-way ANOVA via post-hoc Tukey HDS analysis of embryonal masses. (PDF)

S2 File Two-way ANOVA without replication of shoot-derived tissues. (PDF)

S3 File Pairwise one-way ANOVA via post-hoc Tukey HDS analysis of shoot-derived tissues. (PDF)

S4 File Primer validation via gDNA amplification. (PDF)
