# Supplementary material for: Gene Expression Profiling of Shoot-Derived Calli from Adult Radiata Pine and Zygotic Embryo-Derived Embryonal Masses
Source: PLoS One. 2015 Jun 3;10(6):e0128679. doi: 10.1371/journal.pone.0128679 (PMC4454686; doi:10.1371/journal.pone.0128679)

Pairwise 1-way ANOVA with post-hoc Tukey HDS of embryonal masses

Link to Tukey calculator

Figure 3

YLS8

Tukey HSD results

treatments pair	Tukey HSD Q statistic	Tukey HSD p-value	Tukey HSD inferfence
A vs B	2.4408	0.2710714	insignificant
A vs C	2.6036	0.2351091	insignificant
B vs C	0.1628	0.8999947	insignificant

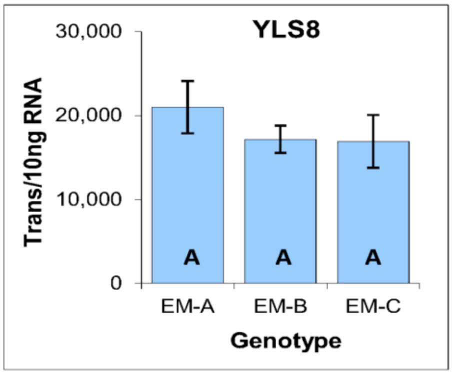

Figure 4

H4

Tukey HSD results

treatments pair	Tukey HSD Q statistic	Tukey HSD p-value	Tukey HSD inferfence
A vs B	2.1258	0.3542583	insignificant
A vs C	5.8322	0.0145336	* p<0.05
B vs C	3.7063	0.0872086	insignificant

PCNA

treatments pair	Tukey HSD Q statistic	Tukey HSD p-value	Tukey HSD inferfence
A vs B	2.3312	0.2978950	insignificant
A vs C	1.0705	0.7294097	insignificant
B vs C	1.2607	0.6585455	insignificant

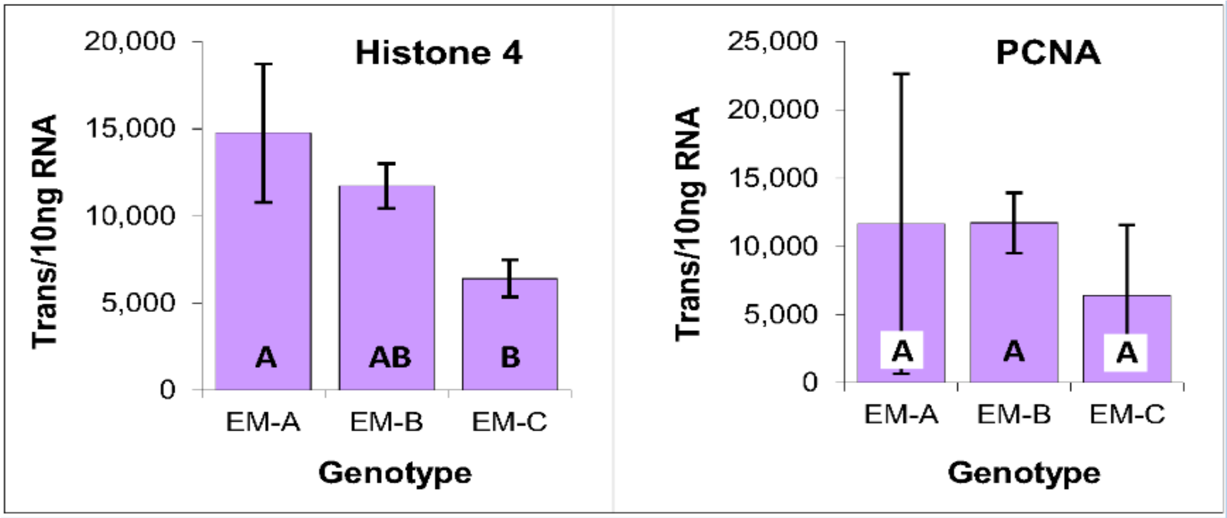

Figure 5

LEC1

treatments pair	Tukey HSD Q statistic	Tukey HSD p-value	Tukey HSD inferfence
A vs B	2.3312	0.2978950	insignificant
A vs C	1.0705	0.7294097	insignificant
B vs C	1.2607	0.6585455	insignificant

WOX2

treatments pair	Tukey HSD Q statistic	Tukey HSD p-value	Tukey HSD inferfence
A vs B	0.6624	0.8814935	insignificant
A vs C	0.3303	0.8999947	insignificant
B vs C	0.3321	0.8999947	insignificant

ABI3

treatments pair	Tukey HSD Q statistic	Tukey HSD p-value	Tukey HSD inferfence
A vs B	3.7329	0.0851491	insignificant
A vs C	0.9991	0.7560265	insignificant
B vs C	2.7338	0.2094958	insignificant

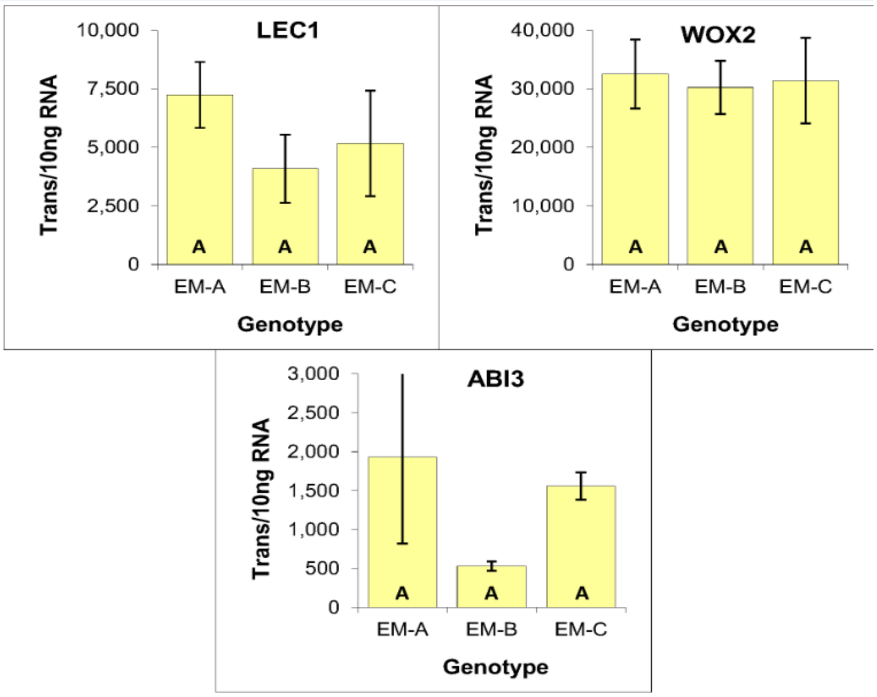

Figure 6

SKN1

treatments pair	Tukey HSD Q statistic	Tukey HSD p-value	Tukey HSD inferfence
A vs B	2.1476	0.3478685	insignificant
A vs C	2.6258	0.2305447	insignificant
B vs C	0.4782	0.8999947	insignificant

SKN2

treatments pair	Tukey HSD Q statistic	Tukey HSD p-value	Tukey HSD inferfence
A vs B	2.6058	0.2346443	insignificant
A vs C	1.0422	0.7399897	insignificant
B vs C	1.5637	0.5456404	insignificant

SKN3

treatments pair	Tukey HSD Q statistic	Tukey HSD p-value	Tukey HSD inferfence
A vs B	0.4983	0.8999947	insignificant
A vs C	0.7973	0.8312185	insignificant
B vs C	0.2990	0.8999947	insignificant

SKN4

treatments pair	Tukey HSD Q statistic	Tukey HSD p-value	Tukey HSD inferfence
A vs B	1.6767	0.5035147	insignificant
A vs C	1.5975	0.5330355	insignificant
B vs C	0.0792	0.8999947	insignificant

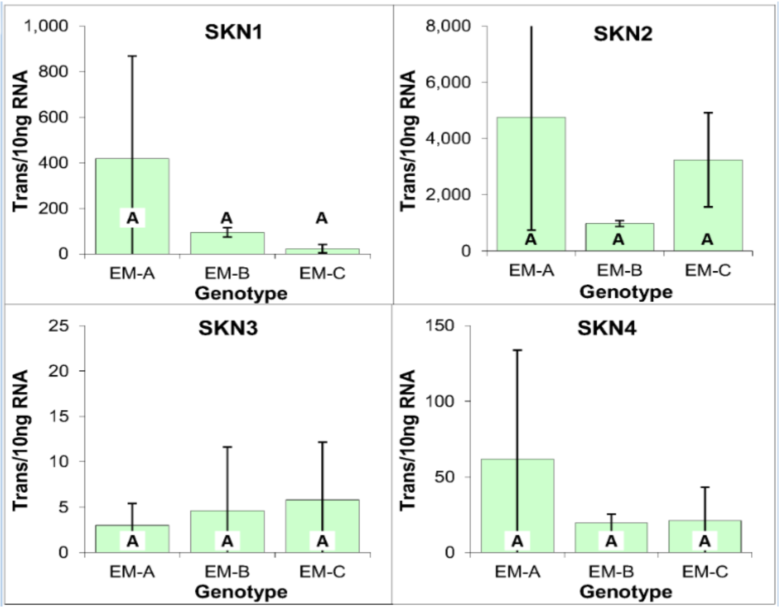

Figure 7
WOX4

treatments pair	Tukey HSD Q statistic	Tukey HSD p-value	Tukey HSD inferfence
A vs B	0.4075	0.8999947	insignificant
A vs C	3.2599	0.1304697	insignificant
B vs C	3.6674	0.0903066	insignificant

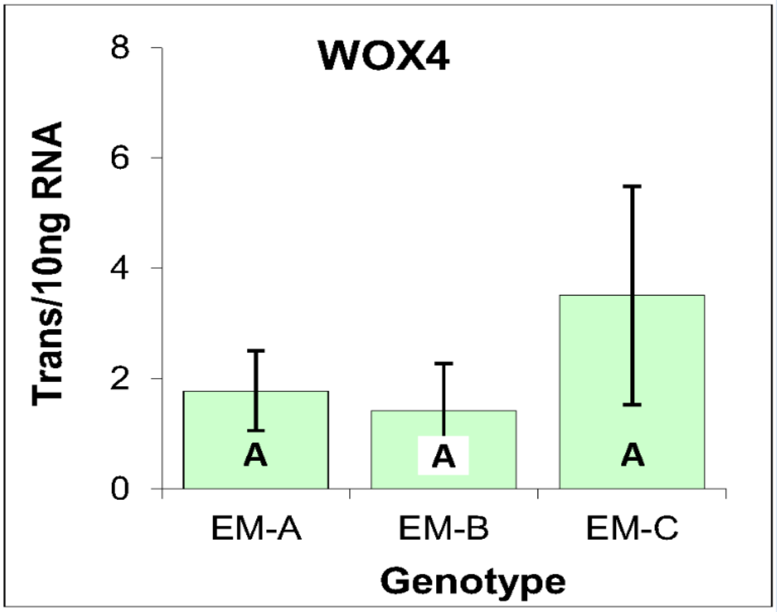

Supplement: S1 File — (PDF) [file pone.0128679.s001.pdf]
