# Supplementary material for: Gene Expression Profiling of Shoot-Derived Calli from Adult Radiata Pine and Zygotic Embryo-Derived Embryonal Masses
Source: PLoS One. 2015 Jun 3;10(6):e0128679. doi: 10.1371/journal.pone.0128679 (PMC4454686; doi:10.1371/journal.pone.0128679)

Excel 2-way ANOVA without replicates of shoot-derived tissues

YLS8

Source of Variation	SS	df	MS	F	P-value	F crit
Rows	16008773	3	5336258	1.929692	0.178659	3.490295
Columns	20260036	4	5065009	1.831603	0.187641	3.259167
Error	33184105	12	2765342			
Total	69452915	19				

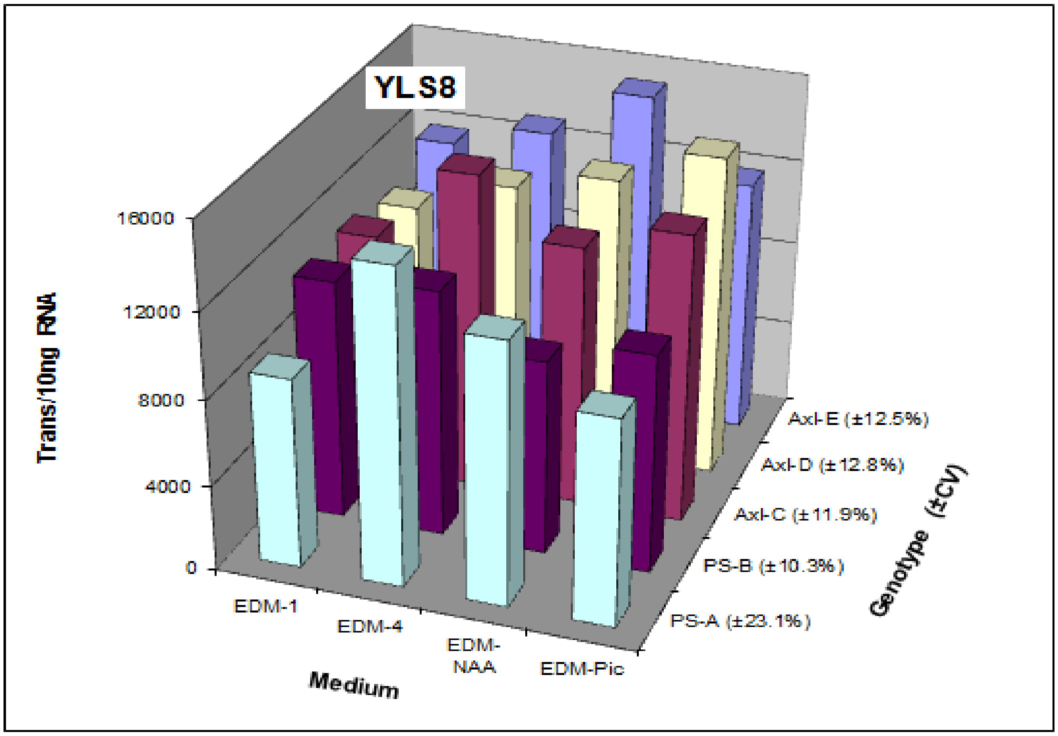

Histone 4

Source of Variation	SS	df	MS	F	P-value	F crit
Rows	14684758	3	4894919	5.058283	0.017126	3.490295
Columns	66525697	4	16631424	17.18648	6.58E-05	3.259167
Error	11612444	12	967703.7			
Total	92822899	19				

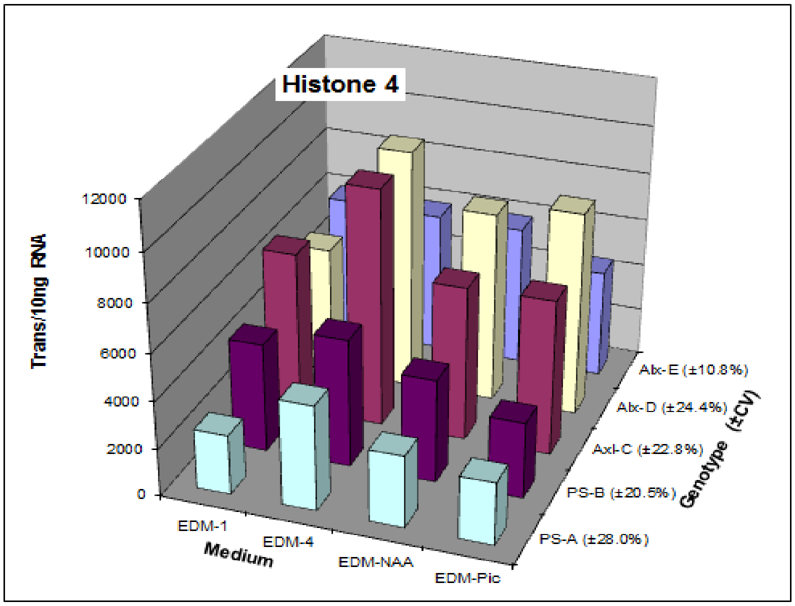

PCNA

Source of Variation	SS	df	MS	F	P-value	F crit
Rows	1.04E+08	3	34653646	3.343437	0.055823	3.490295
Columns	1.78E+09	4	4.45E+08	42.89414	5.16E-07	3.259167
Error	1.24E+08	12	10364677			
Total	2.01E+09	19				

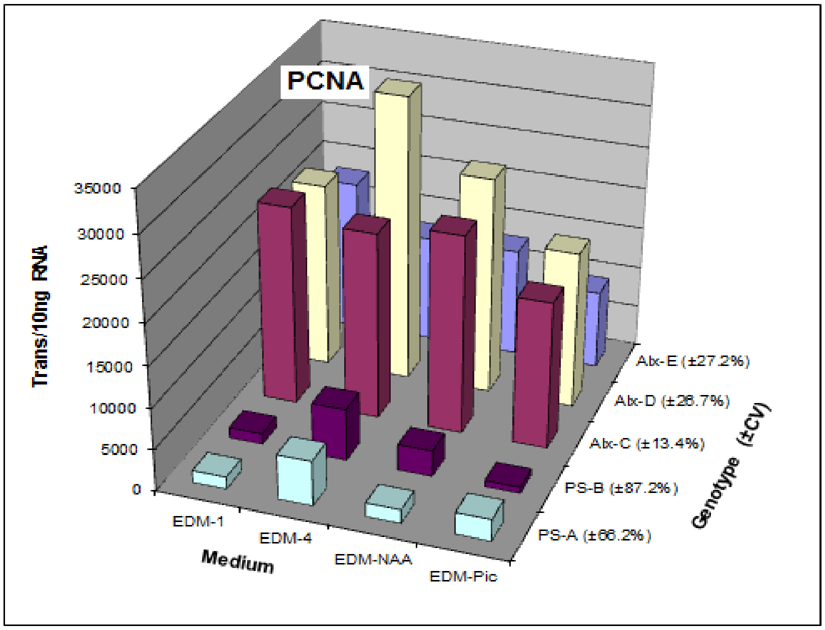

LEC1

Source of Variation	SS	df	MS	F	P-value	F crit
Rows	11357097	3	3785699	4.805206	0.114894	9.276628
Columns	2406641	1	2406641	3.054761	0.178824	10.12796
Error	2363498	3	787832.8			

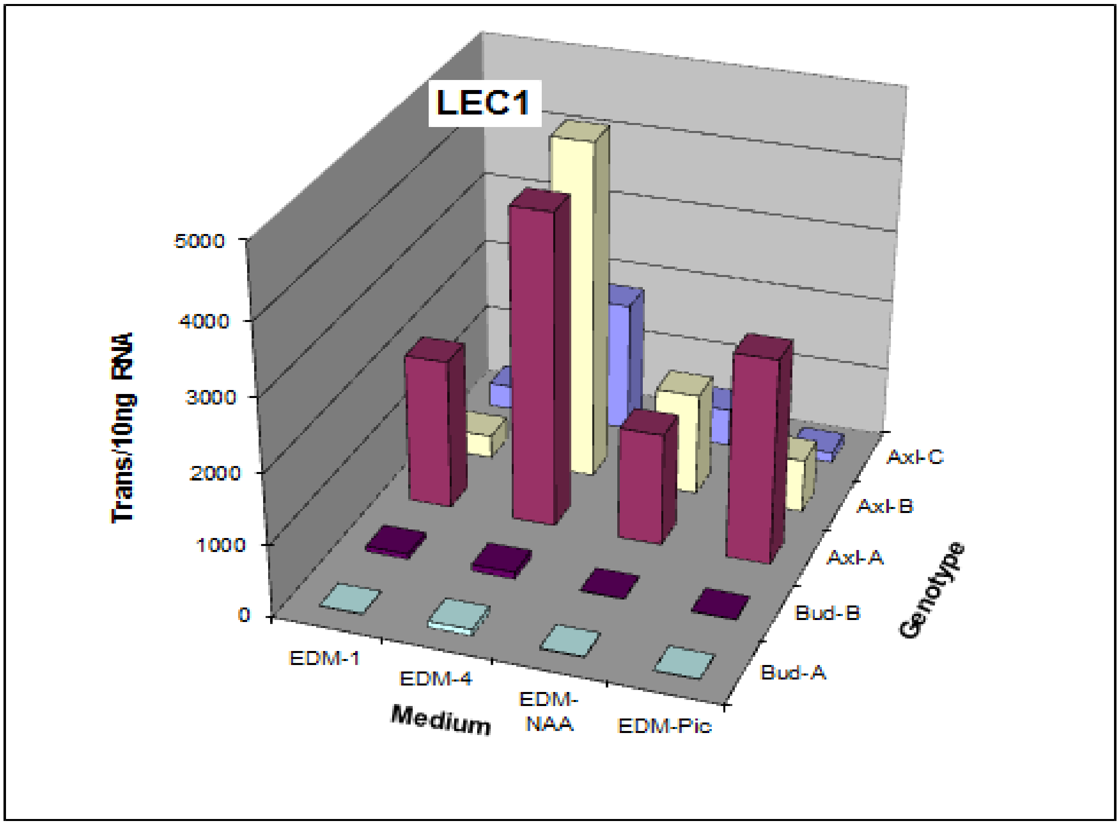

SKN1

Source of Variation	SS	df	MS	F	P-value	F crit
Rows	63313.44	3	21104.48	0.863942	0.486307	3.490295
Columns	80348.65	4	20087.16	0.822297	0.535508	3.259167
Error	293137.3	12	24428.11			
Total	436799.4	19				

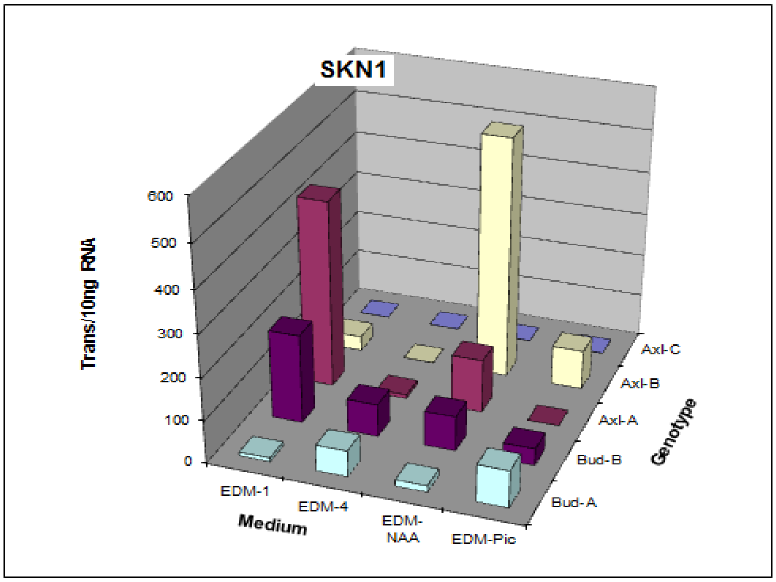

SKN2

Source of Variation	SS	df	MS	F	P-value	F crit
Rows	2936550	3	978850	2.153164	0.146716	3.490295
Columns	13526684	4	3381671	7.43862	0.002973	3.259167
Error	5455320	12	454610			
Total	21918554	19				

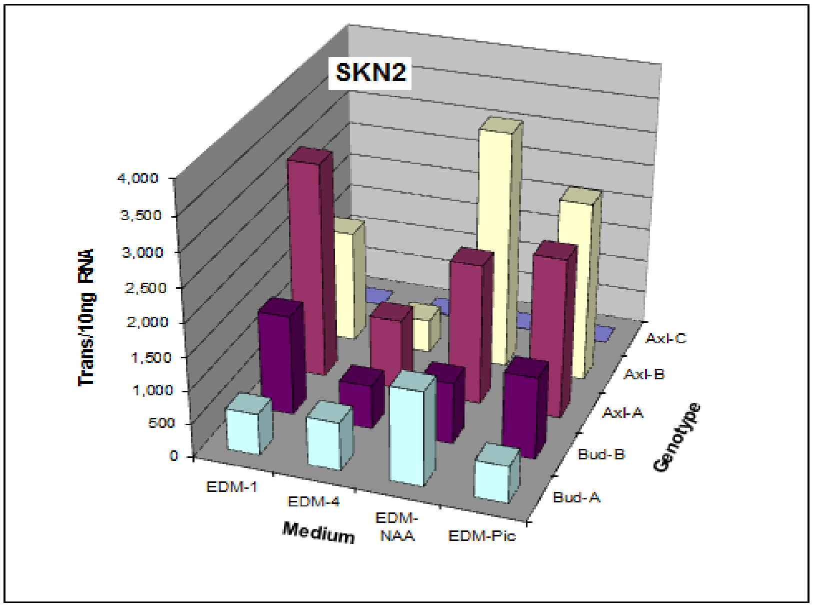

SKN3

Source of Variation	SS	df	MS	F	P-value	F crit
Rows	4911286	3	1637095	1.016036	0.419658	3.490295
Columns	54207330	4	13551833	8.410719	0.001791	3.259167
Error	19335088	12	1611257			
Total	78453705	19				

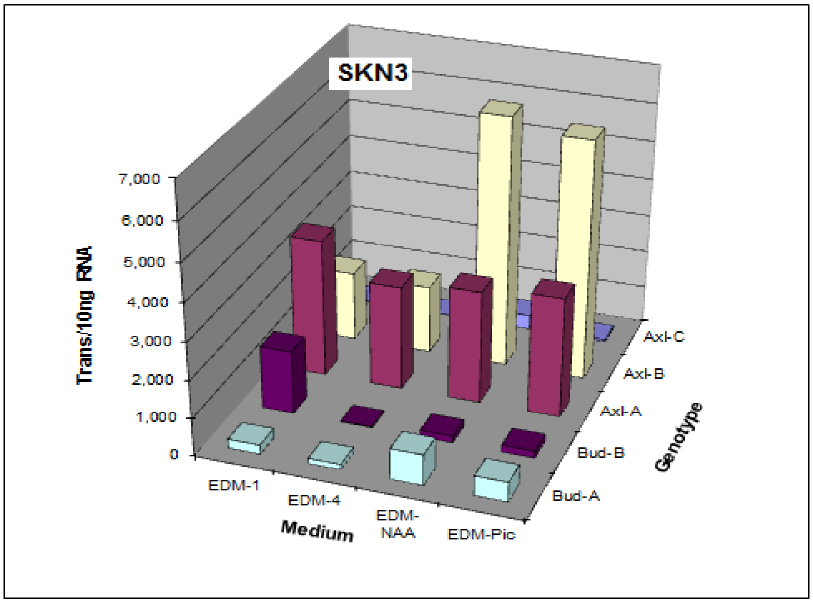

SKN4

Source of Variation	SS	df	MS	F	P-value	F crit
Rows	639513.6	3	213171.2	0.711337	0.563709	3.490295
Columns	3607553	4	901888.2	3.009537	0.061981	3.259167
Error	3596121	12	299676.7			
Total	7843187	19				

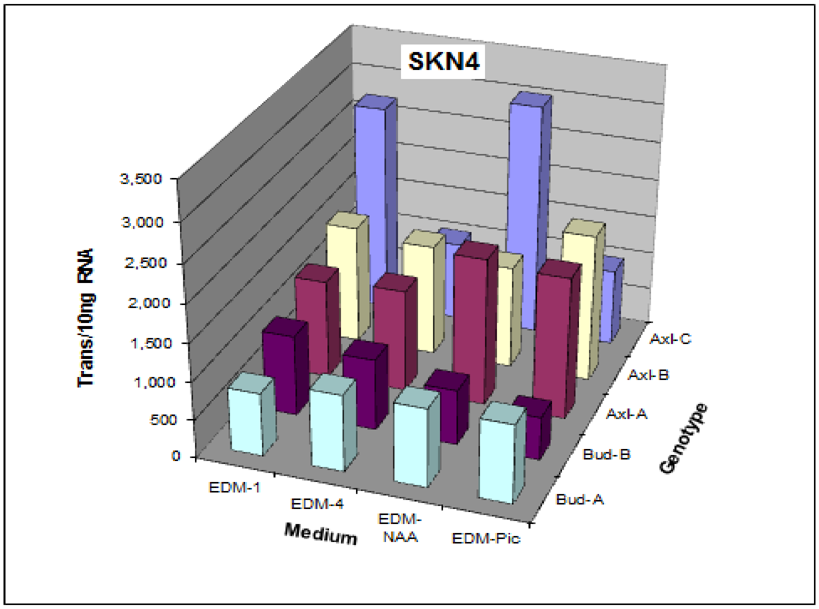

WOX4

Source of Variation	SS	df	MS	F	P-value	F crit
Rows	5995064	3	1998355	1.246175	0.336325	3.490295
Columns	48947408	4	12236852	7.630908	0.00268	3.259167
Error	19243086	12	1603590			
Total	74185557	19				

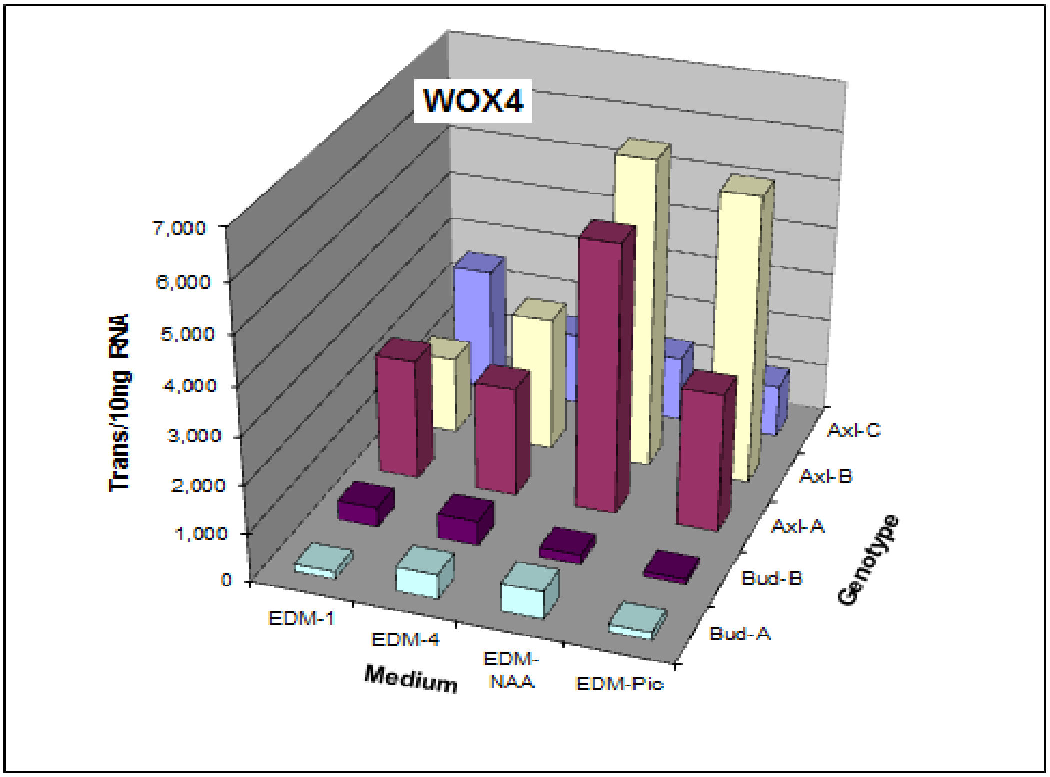

Supplement: S2 File — (PDF) [file pone.0128679.s002.pdf]
