# Supplementary material for: Gene Expression Profiling of Shoot-Derived Calli from Adult Radiata Pine and Zygotic Embryo-Derived Embryonal Masses
Source: PLoS One. 2015 Jun 3;10(6):e0128679. doi: 10.1371/journal.pone.0128679 (PMC4454686; doi:10.1371/journal.pone.0128679)

Pairwise 1-way ANOVA with post-hoc Tukey HDS of shoot-derived tissues

[Link to Tukey calculator]

Figure 8
YLS8

treatments pair	Tukey HSD Q statistic	Tukey HSD p-value	Tukey HSD inferfence
A vs B	0.9931	0.8999947	insignificant
A vs C	1.6616	0.7429291	insignificant
A vs D	1.5561	0.7824727	insignificant
A vs E	1.8046	0.6893107	insignificant
B vs C	2.6547	0.3705671	insignificant
B vs D	2.5492	0.4090182	insignificant
B vs E	2.7977	0.3219152	insignificant
C vs D	0.1055	0.8999947	insignificant
C vs E	0.1430	0.8999947	insignificant
D vs E	0.2485	0.8999947	insignificant

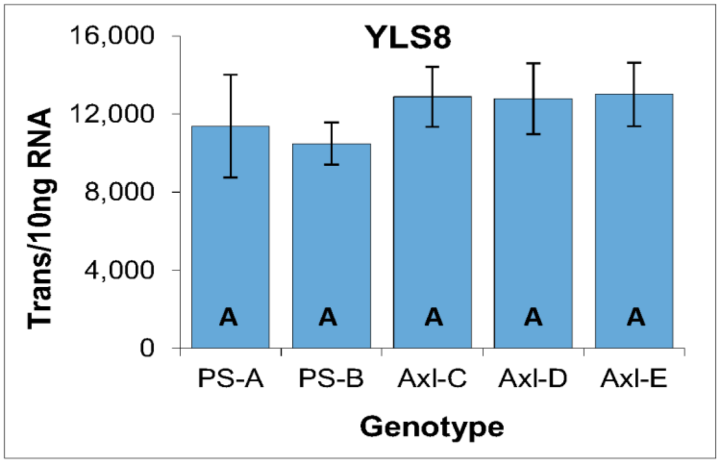

Figure 9
PCNA

treatments pair	Tukey HSD Q statistic	Tukey HSD p-value	Tukey HSD inferfence
A vs B	0.0327	0.8999947	insignificant
A vs C	9.9008	0.0010053	** p<0.01
A vs D	11.6133	0.0010053	** p<0.01
A vs E	5.4395	0.0117421	* p<0.05
B vs C	9.8681	0.0010053	** p<0.01
B vs D	11.5806	0.0010053	** p<0.01
B vs E	5.4068	0.0122802	* p<0.05
C vs D	1.7126	0.7238090	insignificant
C vs E	4.4613	0.0441747	* p<0.05
D vs E	6.1738	0.0042907	** p<0.01

H4

treatments pair	Tukey HSD Q statistic	Tukey HSD p-value	Tukey HSD inferfence
A vs B	1.8527	0.6712826	insignificant
A vs C	6.4968	0.0027618	** p<0.01
A vs D	7.3276	0.0010053	** p<0.01
A vs E	3.6570	0.1236450	insignificant
B vs C	4.6441	0.0346156	* p<0.05
B vs D	5.4749	0.0111876	* p<0.05
B vs E	1.8043	0.6894039	insignificant
C vs D	0.8308	0.8999947	insignificant
C vs E	2.8398	0.3087464	insignificant
D vs E	3.6706	0.1216099	insignificant

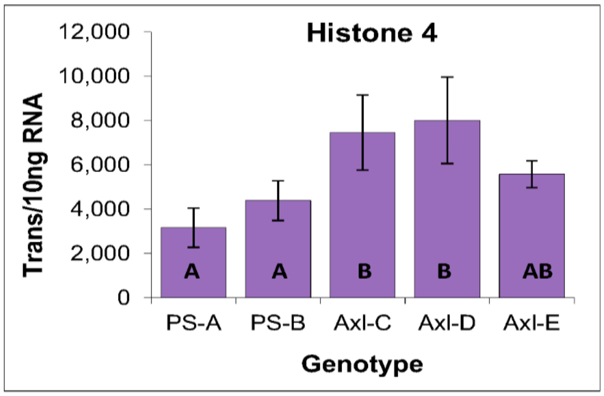

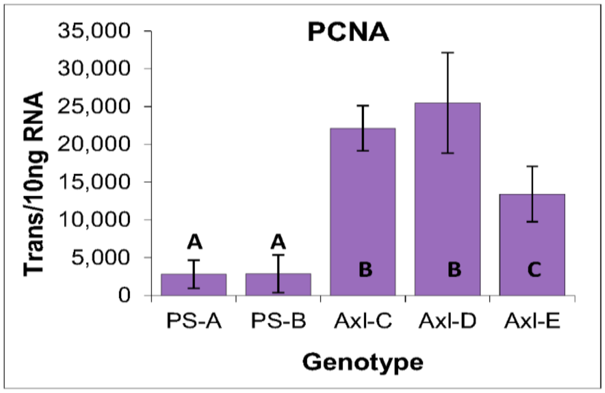

Figure 10

LEC1

treatments pair	Tukey HSD Q statistic	Tukey HSD p-value	Tukey HSD inferfence
A vs B	0.0295	0.8999947	insignificant
A vs C	4.9083	0.0242415	* p<0.05
A vs D	3.2296	0.2036642	insignificant
A vs E	1.2362	0.8999947	insignificant
B vs C	4.8787	0.0252316	* p<0.05
B vs D	3.2001	0.2105220	insignificant
B vs E	1.2066	0.8999947	insignificant
C vs D	1.6786	0.7365265	insignificant
C vs E	3.6721	0.1213817	insignificant
D vs E	1.9935	0.6184919	insignificant

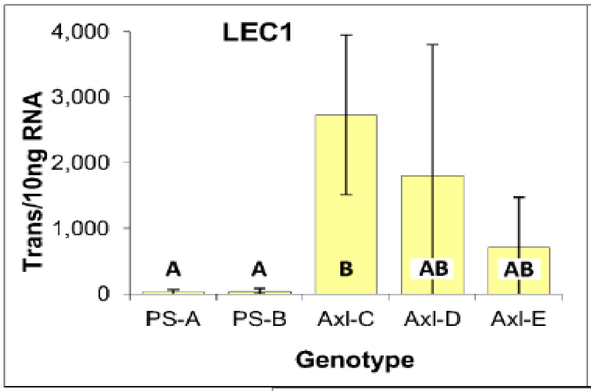

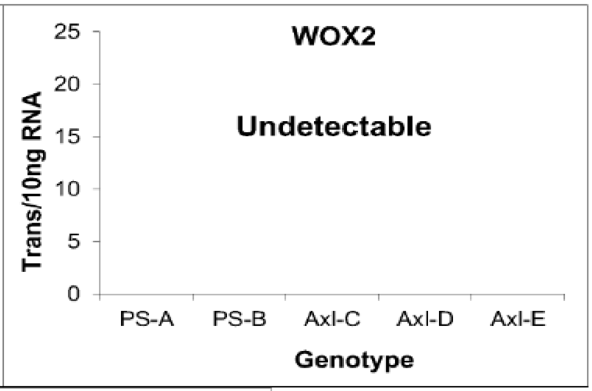

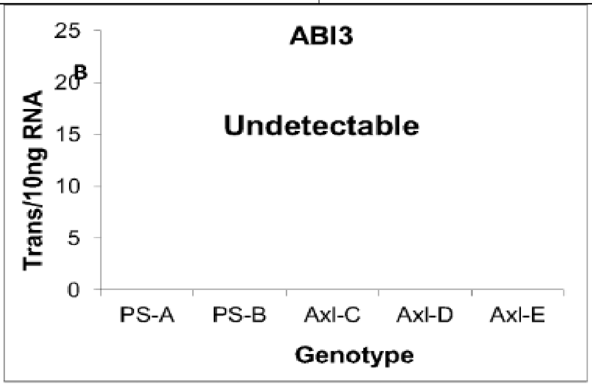

Figure 11

SKN1: Note Axl-E = 0

treatments pair	Tukey HSD Q statistic	Tukey HSD p-value	Tukey HSD inferfence
A vs B	0.6785	0.8999947	insignificant
A vs C	1.1656	0.8283879	insignificant
A vs D	1.4962	0.7037919	insignificant
B vs C	0.4871	0.8999947	insignificant
B vs D	0.8177	0.8999947	insignificant
C vs D	0.3305	0.8999947	insignificant

SKN2

treatments pair	Tukey HSD Q statistic	Tukey HSD p-value	Tukey HSD inferfence
A vs B	0.6651	0.8999947	insignificant
A vs C	3.6589	0.1233591	insignificant
A vs D	3.3975	0.1682093	insignificant
A vs E	2.2091	0.5376466	insignificant
B vs C	2.9938	0.2633489	insignificant
B vs D	2.7325	0.3434966	insignificant
B vs E	2.8742	0.2982186	insignificant
C vs D	0.2613	0.8999947	insignificant
C vs E	5.8680	0.0065200	** p<0.01
D vs E	5.6067	0.0093346	** p<0.01

SKN3

treatments pair	Tukey HSD Q statistic	Tukey HSD p-value	Tukey HSD inferfence
A vs B	0.1290	0.8999947	insignificant
A vs C	4.2615	0.0574855	insignificant
A vs D	5.8294	0.0068759	** p<0.01
A vs E	0.5042	0.8999947	insignificant
B vs C	4.1325	0.0680054	insignificant
B vs D	5.7004	0.0082063	** p<0.01
B vs E	0.6332	0.8999947	insignificant
C vs D	1.5680	0.7780195	insignificant
C vs E	4.7656	0.0293981	* p<0.05
D vs E	6.3336	0.0034491	** p<0.01

SKN4

treatments pair	Tukey HSD Q statistic	Tukey HSD p-value	Tukey HSD inferfence
A vs B	0.5938	0.8999947	insignificant
A vs C	2.3940	0.4678086	insignificant
A vs D	2.2698	0.5149064	insignificant
A vs E	3.6841	0.1196104	insignificant
B vs C	2.9878	0.2650408	insignificant
B vs D	2.8636	0.3014509	insignificant
B vs E	4.2779	0.0562588	insignificant
C vs D	0.1242	0.8999947	insignificant
C vs E	1.2902	0.8821721	insignificant
D vs E	1.4144	0.8356010	insignificant

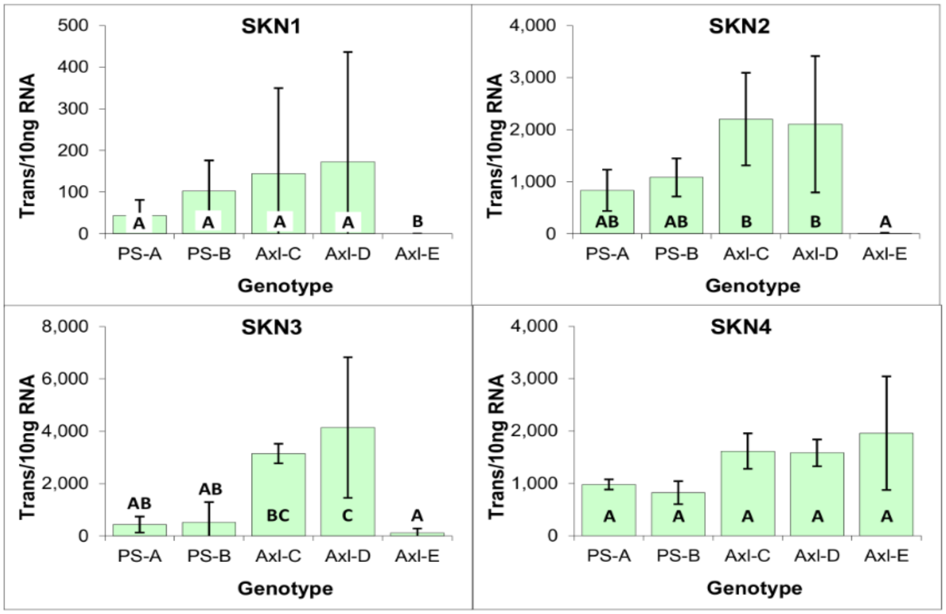

Figure 12
WOX4

treatments pair	Tukey HSD Q statistic	Tukey HSD p-value	Tukey HSD inferfence
A vs B	0.0736	0.8999947	insignificant
A vs C	4.5736	0.0380453	* p<0.05
A vs D	5.9126	0.0061336	** p<0.01
A vs E	1.9792	0.6238433	insignificant
B vs C	4.6472	0.0344754	* p<0.05
B vs D	5.9862	0.0055456	** p<0.01
B vs E	2.0528	0.5962431	insignificant
C vs D	1.3390	0.8638604	insignificant
C vs E	2.5944	0.3923494	insignificant
D vs E	3.9334	0.0875581	insignificant

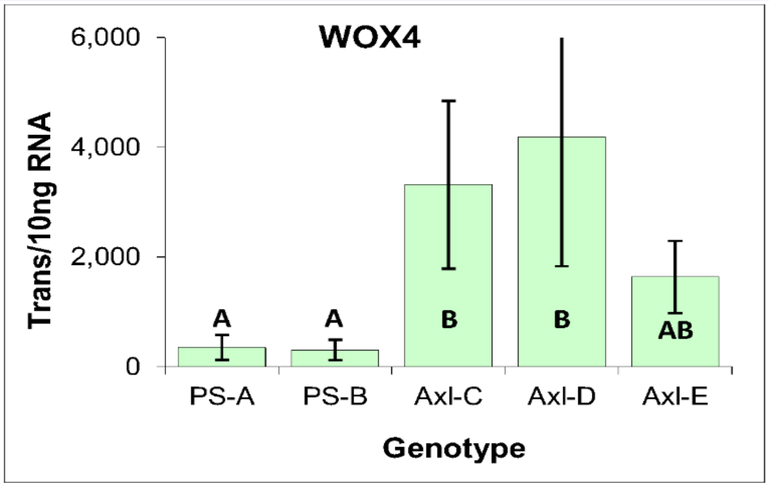

Supplement: S3 File — (PDF) [file pone.0128679.s003.pdf]
