# Supplementary material for: Gene Expression Profiling of Shoot-Derived Calli from Adult Radiata Pine and Zygotic Embryo-Derived Embryonal Masses
Source: PLoS One. 2015 Jun 3;10(6):e0128679. doi: 10.1371/journal.pone.0128679 (PMC4454686; doi:10.1371/journal.pone.0128679)
Supplement: S4 File — (PDF) [file pone.0128679.s004.pdf]

Primer validation via gDNA amplification

To confirm absence of polymorphic sequences that significantly disrupt primer annealing and elongation, each of the primer pairs was used to amplify 1 ng of gDNA isolated from each of the genotypes and the resulting quatities converted to copies/genome based on a haploid genome size of 22.25 pg/genome (41C/ng).

Note that the central objective was to confirm similarities in the relative quantities across the genotypes rather than to attempt to precisely determine the number of genes per genome.

A publication is currently being prepared that describes this approach in detail.
Contact R.G. Rutledge for additional details.

Copies per Genome

	YLS8	Histone 4	PCNA	LEC1	WOX2	ABI3	SKN1	SKN2	SKN3	SKN4	WOX4
PS-A	3.61	1.41	1.16	1.75	1.33	0.95	0.95	1.24	1.21	0.99	1.09
PS-B	3.03	1.67	1.33	1.36	0.85	0.90	1.07	1.02	1.53	0.99	1.29
Axl-C	2.91	1.84	1.62	1.89	1.19	0.65	1.19	0.92	0.90	0.87	1.19
Axl-D	2.55	1.38	0.95	1.62	1.12	0.51	0.95	0.70	0.97	1.09	0.99
Axl-E	2.80	1.06	0.92	1.74	1.03	0.99	1.02	0.82	0.89	0.75	0.85
**Average:**	2.98	1.47	1.20	1.67	1.10	0.80	1.03	0.94	1.10	0.94	1.08
**CV:**	13.3%	20.3%	24.4%	11.9%	16.4%	25.9%	9.7%	21.6%	24.8%	13.9%	15.5%
**Amplion Size:**	172	163	154	189	223	82	116	186	151	203	207
